# Myxoma virus lacking the host range determinant M062 stimulates cGAS-dependent type 1 interferon response and unique transcriptomic changes in human monocytes/macrophages

**DOI:** 10.1371/journal.ppat.1010316

**Published:** 2022-09-14

**Authors:** Steven J. Conrad, Tahseen Raza, Erich A. Peterson, Jason Liem, Richard Connor, Bernice Nounamo, Martin Cannon, Jia Liu

**Affiliations:** 1 Department of Microbiology and Immunology, University of Arkansas for Medical Sciences (UAMS), Little Rock, Arkansas, United States of America; 2 Winthrop P. Rockefeller Cancer Institute, University of Arkansas for Medical Sciences, Little Rock, Arkansas, United States of America; 3 Center of Pathogenesis and Host Inflammatory Responses, University of Arkansas for Medical Sciences (UAMS), Little Rock, Arkansas, United States of America; University of New Mexico, UNITED STATES

## Abstract

The evolutionarily successful poxviruses possess effective and diverse strategies to circumvent or overcome host defense mechanisms. Poxviruses encode many immunoregulatory proteins to evade host immunity to establish a productive infection and have unique means of inhibiting DNA sensing-dependent type 1 interferon (IFN-I) responses, a necessity given their dsDNA genome and exclusively cytoplasmic life cycle. We found that the key DNA sensing inhibition by poxvirus infection was dominant during the early stage of poxvirus infection before DNA replication. In an effort to identify the poxvirus gene products which subdue the antiviral proinflammatory responses (e.g., IFN-I response), we investigated the function of one early gene that is the known host range determinant from the highly conserved poxvirus host range *C7L* superfamily, myxoma virus (MYXV) M062. Host range factors are unique features of poxviruses that determine the species and cell type tropism. Almost all sequenced mammalian poxviruses retain at least one homologue of the poxvirus host range *C7L* superfamily. In MYXV, a rabbit-specific poxvirus, the dominant and broad-spectrum host range determinant of the *C7L* superfamily is the *M062R* gene. The *M062R* gene product is essential for MYXV infection in almost all cells tested from different mammalian species and specifically inhibits the function of host Sterile α Motif Domain-containing 9 (SAMD9), as *M062R*-null (Δ*M062R*) MYXV causes abortive infection in a SAMD9-dependent manner. In this study we investigated the immunostimulatory property of the Δ*M062R*. We found that the replication-defective Δ*M062R* activated host DNA sensing pathway during infection in a cGAS-dependent fashion and that knocking down SAMD9 expression attenuated proinflammatory responses. Moreover, transcriptomic analyses showed a unique feature of the host gene expression landscape that is different from the dsDNA alone-stimulated inflammatory state. This study establishes a link between the anti-neoplastic function of SAMD9 and the regulation of innate immune responses.

## Author summary

Poxviruses encode a group of genes called host range determinants to maintain or expand their host tropism. The mechanism by which many viral host range factors function remains elusive. Some host range factors possess immunoregulatory functions responsible for evading or subduing host immune defense mechanisms. Most known immunoregulatory proteins encoded by poxviruses are dispensable for viral replication *in vitro*. The uniqueness of MYXV *M062R* is that it is essential for viral infection in vitro and belongs to one of the most conserved poxvirus host range families, the *C7L* superfamily. There is one known host target of the MYXV M062 protein, SAMD9. SAMD9 is constitutively expressed in mammalian cells and exclusively present in the cytoplasm with an anti-neoplastic function. Humans with deleterious mutations in SAMD9 present disease that ranges from lethality at a young age to a predisposition to myelodysplastic syndromes (MDS) that often require bone marrow transplantation. More importantly, SAMD9 serves as an important antiviral intrinsic molecule to many viruses. The cellular function of SAMD9 remains unclear mostly due to the difficulty of studying this protein, i.e., its large size, long half-life, and its constitutive expression in most cells. In this study we used *M062R*-null MYXV as a tool to study SAMD9 function and report a functional link between SAMD9 and the regulation of the proinflammatory responses triggered by cGAS-dependent DNA sensing.

## Introduction

Mammalian hosts have sophisticated regulation for the triggering of pro-inflammatory responses, especially after detecting danger signals in the cytoplasm. Many fundamental sensing instruments and their direct downstream signaling axes have been described, such as cGAS/STING/IRF3 axis for DNA sensing [1–3] and RNA sensing pathways [4] e.g., the RIG-I/MAVS/IRF3 axis [5]. Additional factors may fine-tune the consequences of dangerous stimuli (e.g., DNA substrates) resulting in distinctive overall cellular and immunological responses. The outcome may also be tissue- and cell-type dependent. We are particularly interested in understanding the immunoregulatory mechanism of monocytes/macrophages. These immune cells are among the first responders to viral infection and also important in the maintenance of immunological microenvironment, such as the tumor environment.

Cytoplasmic surveillance for the presence of DNA is an important task for mammalian cells, as the appearance of DNA in this privileged compartment signals grave danger for the well-being of the cell. Poxviruses, especially virulent poxviruses, must inhibit the host DNA sensing pathway and IFN-I production to be able to effectively replicate and spread to other cells [6]. Many orthopoxviruses encode a poxvirus immune nuclease (poxin), an early poxvirus gene, for cGAS-STING-specific immune evasion [7–10], but poxviruses from non-orthopoxvirus genera may not possess such genes in their genomes. One such example is MYXV. It was reported previously that the gene product of *F17R*, a late gene, from vaccinia virus (VACV) was important for inhibiting DNA sensing [11,12] and a homolog of the VACV *F17R* is present in MYXV, *M026R* ([13]. Poxviruses may inhibit DNA sensing at an early time during infection and poxviruses from different genera likely have evolved unique strategies to circumvent host surveillance against cytoplasmic DNA. Here we investigated such phenomenon and its associated transcriptomic remodeling in macrophages using a mutant MYXV with targeted deletion in *M062R* gene, Δ*M062R*. Although it triggers the type 1 interferon (IFN-I) response through activation of the DNA sensing pathway, we found the response by Δ*M062R* distinct from that caused by the classic dsDNA sensing alone. This is most likely due to the fact that Δ*M062R* induced proinflammatory effect is also regulated by another host protein, SAMD9. This unusual inflammatory response induced by Δ*M062R* may explain the immunotherapeutic benefit we have observed when using Δ*M062R* in the tumor environment and when treating tumor associated macrophages (TAMs) from human patients [14]. Treatment with Δ*M062R* turns off the typical immunosuppressive characteristics in TAMs which in turn restores and promotes helper T cell function against tumor antigen in the tumor environment.

Poxviruses are exemplary probing tools in our quest to understand the host immune response(s) at the molecular and pathogenesis levels. Poxvirus must be able to evade host surveillance against cytoplasmic DNA due to their exclusive cytoplasmic life cycle to successfully replicate their dsDNA genome. It is not surprising that many genes encoded by poxviruses antagonize DNA sensing-stimulated antiviral immune responses.

In this study we utilized MYXV, a rabbit-specific poxvirus, to investigate novel host regulation of innate immune responses using a viral protein, M062, as a probing tool. Myxoma virus (MYXV) belongs to the genus of *Leporipoxvirus* and has a narrow host tropism in lagomorphs with natural hosts in *Sylvilagus* species [15] and is an excellent model organism to study poxvirus evolution in the susceptible hosts [16]; MYXV is also a promising oncolytic candidate for cancer therapy [17,18]. Despite its narrow host tropism, MYXV is able to evade many cellular defense mechanisms in a species-independent manner [19–22], including dsRNA-dependent translation inhibition [23]. It was shown that in human primary macrophages, wildtype MYXV cannot evade host RNA sensing mechanism such as that through RIG-I [24], but it is not known if MYXV is able to evade DNA sensing in a species-independent manner, which is the focus of this study. Viral M062 protein is essential for MYXV infection and the *M062R* gene belongs to the poxvirus host range *C7L* superfamily [15]. In MYXV genome, unlike most other mammalian poxviruses, there are 3 *C7L* homologs, *M062R*, *M063R*, and *M064R* [25] with *M062R* the dominant and broad-spectrum host range determinant [26]. M062 has a known host target, SAMD9, and inhibition of SAMD9 is required for a productive viral infection [26,27]. We observed that infection by the replication-defective Δ*M062R* in monocytes/macrophages led to IFN-I induction and the production of proinflammatory cytokines/chemokines. This proinflammatory response-associated host gene expression is IRF-dependent and is regulated through DNA sensing by cGAS. The proinflammatory responses caused by Δ*M062R* are also regulated by SAMD9, the direct target of viral M062 protein. Interestingly, this dual regulation leads to a unique transcriptomic landscape distinct from what is induced by dsDNA sensing alone. We thus concluded that this additional regulation of the cGAS DNA sensing pathway through SAMD9 may act to fine-tune the consequences of DNA sensing. This finding elucidates the immunoregulatory function of SAMD9 in addition to its anti-neoplastic property and may explain the role of SAMD9 in host defense against cytoplasmic danger signals.

## Results

### Early poxvirus proteins play dominant roles in suppressing dsDNA-stimulated IFN-I production

**Fig 1:**
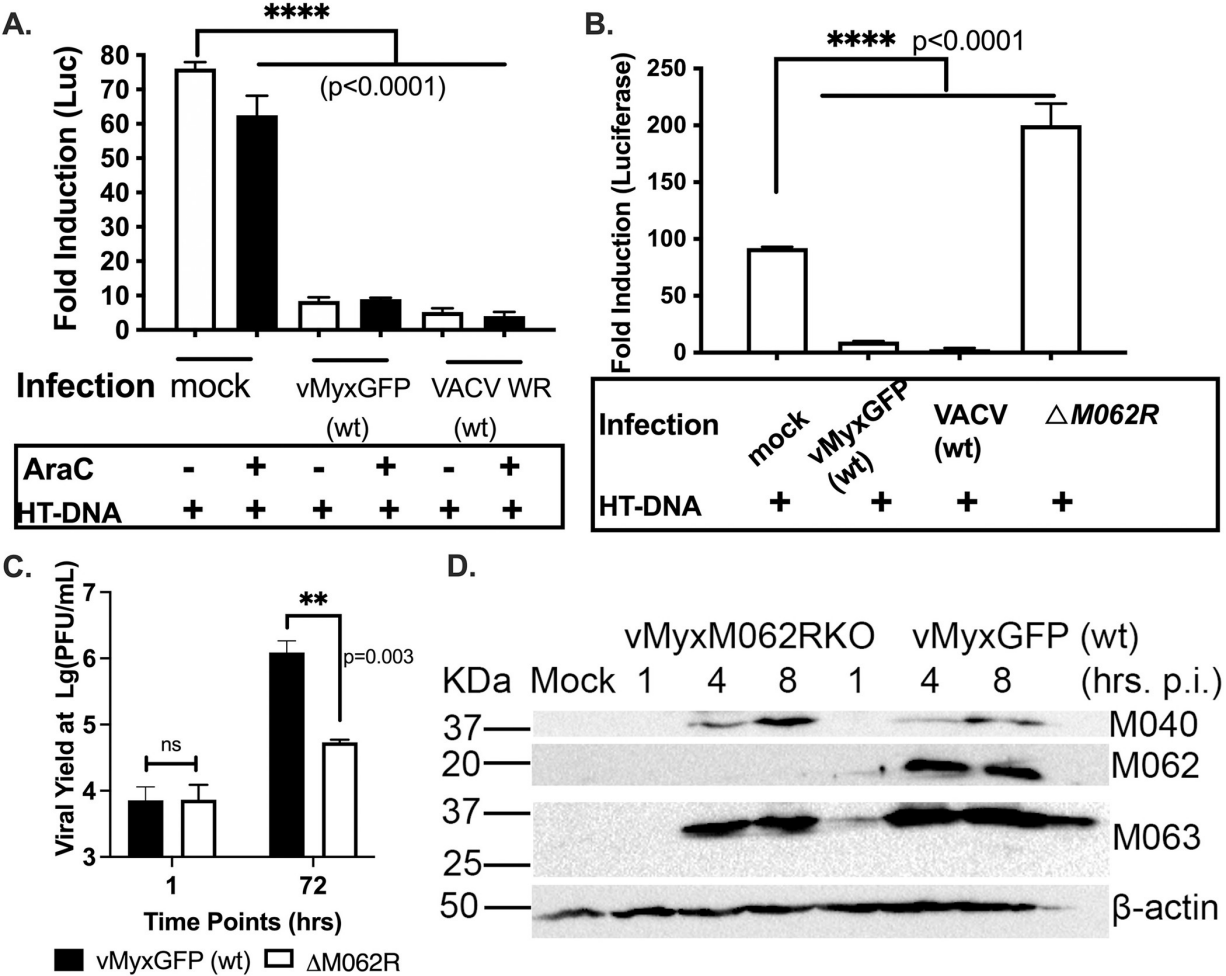
Myxoma virus *M062R* gene is important for viral inhibition of DNA sensing. **A.** Early gene expression from either wildtype MYXV or VACV inhibits dsDNA-stimulated IFN-I induction. The IRF-dependent luciferase-expressing THP-1 cell line is a surrogate system for IFN-I induction. After cells were differentiated into macrophage-like cells, viral infection was performed at an moi of 2 for VACV and 10 for MYXV in the presence of AraC (100 μM) for 12 hours (hrs) before cells were transfected with HT-DNA (herring testis DNA) at 0.3 μg/1million cells for 18 hrs. In the absence of post-replicative gene products due to the AraC treatment, wildtype MYXV or VACV infection significantly inhibited dsDNA-stimulated luciferase expression. Statistical analyses were performed with ordinary one-way ANOVA followed by Tukey’s multiple comparison test and p<0.05 is defined as being statically significant (****p<0.0001). Shown is a representative result of 2 biological replicates and each data point is an average of triplicate measurements (technical replicates). **B.** In the absence of *M062R* gene, the resulting ΔM*062R* MYXV loses the ability to inhibit HT-DNA stimulated luciferase expression. The same luciferase-expressing THP-1 cells were differentiated into macrophages as in “**A**”. After viral infection at a high moi of 2 with VACV or 10 with MYXV for 12 hrs, cells were transfected with HT-DNA for 18 hrs. Supernatant was then collected to measure the luciferase activities. *M062R*-knockout MYXV could no longer inhibit dsDNA-stimulated IRF-dependent luciferase expression. Statistical analyses were performed using ordinary one-way ANOVA followed by Dunnett multiple comparison test and p<0.05 is defined as being statistically significant (****p<0.0001). Shown is a representative of 3 biological replicates and each data point is the average of 2 replicating measurements. **C**. Infection by Δ*M062R* MYXV leads to abortive infection. Similar to the majority cells tested (26), THP1 differentiated macrophage-like cells are infected by either WT or Δ*M062R* MYXV at an moi of 1. At 1 and 72 hrs post-infection, cells were harvested for titration on BSC-40 cells. Triplicate for infection at each given time point for each virus are performed and titration is also performed in triplicate at each dilution. Shown is a representative of 2 independent experiments. Two-way ANOVA multiple comparison is performed and statistical significance is defined as p<0.05 and ** for p<0.01. **D.** Infection by ΔM*062R* MYXV leads to comparable early gene expression as WT infection. Differentiated THP1 cells are infected with either ΔM*062R* or WT MYXV at a moi of 5 and cell lysates were harvested at the given time point for western blot. A total protein of 30 μg is loaded per sample, early viral proteins such as M040 (VACV I3 homolog) and M063 are probed. The expression of M062 is shown for the WT infection. Consistent with a previous report (26), Δ*M062R* infection in differentiated THP1 cells does not disrupt early gene expression.

Our initial experiments aimed to rule out the possibility that any early viral genes from MYXV might play a role in the inhibition of DNA sensing with VACV strain Western Reserve (WR) as a control. We utilized human monocytic THP-1 cells expressing luciferase for the study. THP-1 cells can be differentiated into macrophages for testing DNA sensing and downstream outcome, and the firefly luciferase (F-Luc) expression is driven by the IRF recognition domain (Invivogen, San Diego, CA) [28]. Using this well-established IRF-dependent luciferase system in macrophages in the presence of a DNA replication inhibitor, cytosine arabinoside (AraC), we found MYXV infection at the early stage could already potently inhibit dsDNA-stimulated (herring testis dsDNA or HT-DNA) luciferase expression comparably to the ability by VACV (**Fig 1A**). The levels of inhibition in the presence of AraC from both VACV and MYXV are similar to those caused by corresponding viruses without AraC treatment (**Fig 1A**). We confirmed the AraC treatment effect in inhibiting post-replicative gene expression through monitoring the fluorescent protein expression driven by late (tdtomato red or tdTred in the wildtype VACV) [29] and early/late (GFP in the wildtype MYXV) [26,30] poxvirus promoters (Supplemental **Fig 1A** and **1B**). For instance, at the time of testing in Fig 1A, late promoter-driven tdTred were not detected in wildtype VACV infected cells (**S1A Fig**) before the luciferase assay, and early/late promoter driven GFP from wildtype MYXV (**S1B Fig**) is also significantly reduced compared to no AraC treatment with the same corresponding infections. With western blot (**S1C Fig**), under the AraC dose used in Fig 1A and 1B, we no longer detect MYXV intermediate protein M038 (a homolog of VACV I1) and late protein SERP1. We chose a lower dose of AraC to avoid toxic and DNA-sensing stimulatory effects [31] on differentiated THP1 cells, and such dosage obtained a similar effect in inhibiting post-replicative gene expression to what we typically used 200mM dose in other studies [19,26]. Thus, additional viral factors that are expressed during early gene expression play dominant roles in the suppression of DNA-induced IFN-I induction in MYXV. Next, we found that infection by a replication defective virus with the essential host range gene *M062R* deleted (**Fig 1C**), Δ*M062R*, lost the ability to inhibit dsDNA-stimulated IFN-I induction (**Fig 1B**). It is known that Δ*M062R* infection retains early viral gene expression, e.g., M040, a homolog of VACV I3, and M063, comparable to what is seen in the wildtype MYXV infection (**Fig 1D**) and [26]. In conclusion, instead of what we thought originally, early genes of VACV and MYXV play the important role for the inhibition of host DNA sensing.

### *M062R*-null MYXV (Δ*M062R*) infection stimulates proinflammatory cytokine production

**Fig 2:**
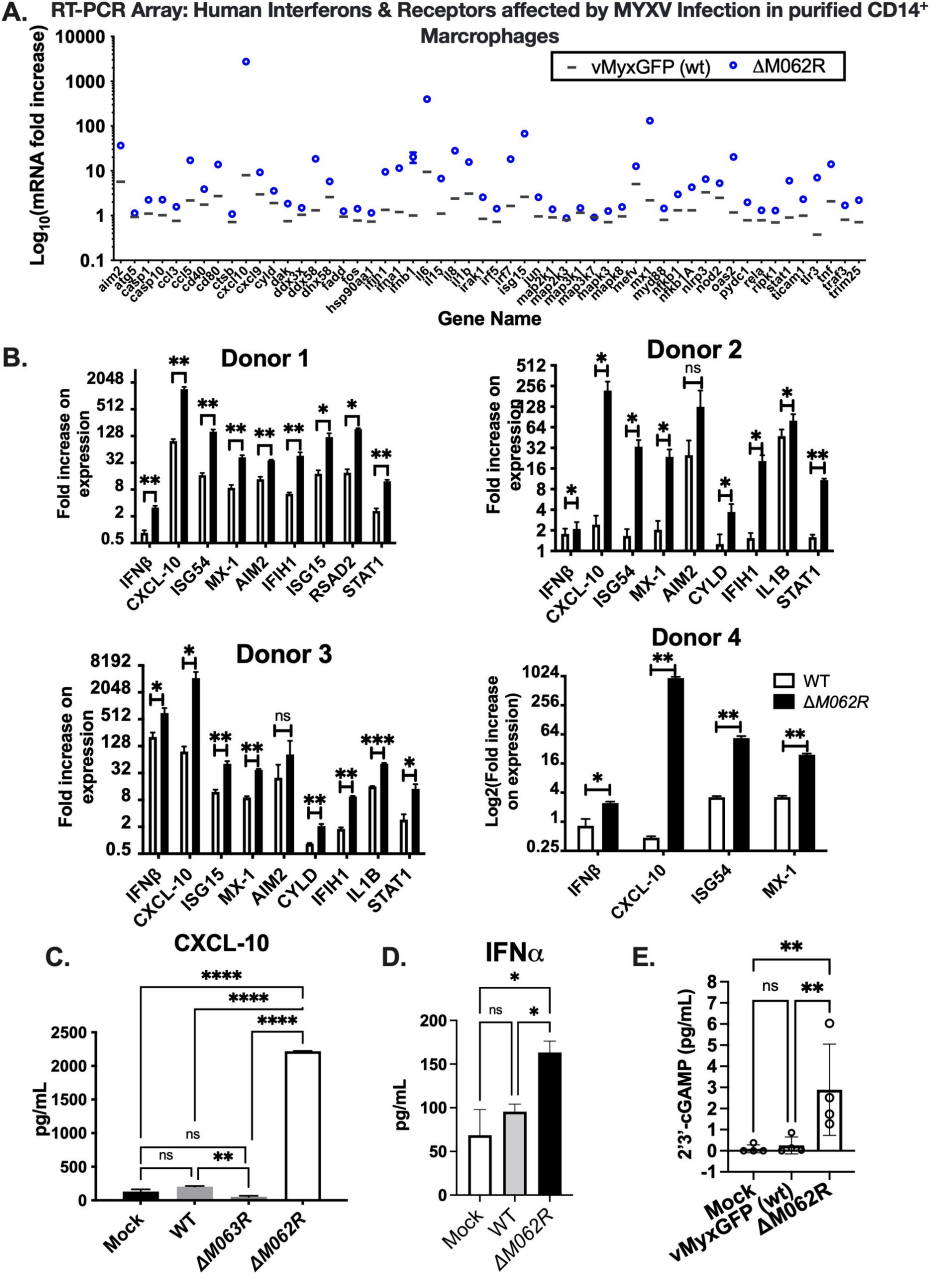
Infection by Δ*M062R* MYXV stimulates the expression of interferon-stimulated genes (ISGs). **A**. Infection by Δ*M062R* MYXV stimulates higher levels of IFN and ISG RNAs in primary human CD14^+^ monocytes than that from the wildtype MYXV infection. Infection by MYXV, wildtype and Δ*M062R*, was performed at an moi of 10 and cells were collected at 16 hours post-infection (p.i.) for RNA extraction, reverse transcription, and RT-PCR according to the manufacturer’s protocol. The ΔCt and ΔΔCt was calculated and RNA levels were normalized to an internal control gene, B2M. By normalizing to the result of the mock infected group for the same gene, fold induction of the corresponding gene is calculated using the formula of Foldchange = 2^(-ΔΔCt). Shown is representative data from one of two healthy donors. **B**. Using RT^2^-PCR we confirmed that ΔM*062R* led to upregulation of IFN-I and pro-inflammatory molecules in human CD14^+^ primary monocytes. Primary CD14^+^ human monocytes from 4 healthy human donors were mock infected or infected with either wildtype or Δ*M062R* MYXV. In each case the expression of mRNAs encoding several ISGs including CXCL-10, ISG54 and MX-1 were elevated in the monocytes/macrophages infected with ΔM*062R* MYXV compared to monocytes/macrophages infected with wildtype MYXV. Fold changes are measured by normalizing to that of mock infection. Statistical analyses of one sample t and Wilcoxon test are performed with statistical significance as *p<0.05, **p<0.01, and ***p<0.001. **C**. Infection by ΔM*062R* stimulates the production of CXCL10. As a mutant virus control the *M063R*-null MYXV (Δ*M063R*) was used to infect primary CD14^+^ human monocytes along with other controls (mock infection and wildtype MYXV or WT) and experimental group of ΔM*062R* infection. CXCL-10 levels in the supernatant is shown and the Δ*M062R*-infected monocytes/macrophages secreted significantly higher levels of CXCL-10 than the control groups. Statistical analysis of ordinary one-way ANOVA and Tukey’s multiple comparison test are performed. Statistical significance is defined as **p<0.01, **p<0.001, and ****p<0.0001. **D** Infection by ΔM*062R* stimulated interferon α (IFNα). Primary CD14^+^ human monocytes are mock treated, infected by WT or Δ*M062R* for 16 hrs. Supernatant is collected for detection of IFNα. Statistical analysis of ordinary one-way ANOVA and multiple comparison are performed. The statistical significance is defined as *p<0.05. **E.** Infection by ΔM*062R* in THP-1 derived macrophages induces 2’3’-cGAMP. THP1 macrophages are mock treated, infected with wildtype MYXV, or infected with ΔM*062R* at an moi of 5 for 8 hours before supernatant is harvested for 2’3’-cGAMP ELISA. Shown is the representative data from 2 biological replicates with 4 technical replicates per sample. Statistical analysis is performed with Ordinary one-way ANOVA and multiple comparisons. Statistical significance is defined as *p<0.05 and **p<0.01.

The MYXV early gene, *M062R*, is a broad-spectrum host range determinant from the poxvirus host range *C7L* superfamily [15] and is essential for MYXV infection [26,27]. The Δ*M062R* MYXV has a tropism defect and causes an abortive infection in almost all cells tested from species such as humans and rabbits [26]. During Δ*M062R* infection both viral DNA replication and late protein synthesis are significantly inhibited resulting in an abortive infection [26] (Fig 1C), while early gene expression remains intact (**Fig 1D**). The virotherapeutic benefit we observed prompted us to investigate the immunological effect caused by Δ*M062R* [32], since this mutant virus is unable to establish a productive viral replication and the oncolytic effect of Δ*M062R* is not through direct induction of cell death, e.g., apoptosis. We performed a RT^2^ profiler screening for antiviral responses to compare how responses generated by Δ*M062R* infection differed from the wildtype MYXV infection. We found Δ*M062R* infection in human primary monocytes stimulated the expression of many antiviral and interferon-stimulated genes (ISGs) (**Fig 2A**). To validate the above findings from the screening we collected peripheral blood from 4 healthy individuals and purified CD14^+^ monocytes/macrophages for validation. We found Δ*M062R* infection in these cells generally stimulated elevated IFN-I and ISGs, e.g., IFN β and CXCL-10 (**Fig 2B**), consistent with the previous screening results. We also confirmed the elevated CXCL-10 and IFNα levels in the supernatant of Δ*M062R* infected cells (**Fig 2C** and **2D**, respectively). As a control we included a MYXV deletion mutant in which another *C7L* superfamily gene, *M063R*, was ablated, *M063R*-null MYXV [33] (Δ*M063R*). *M063R*-null MYXV remains replication-competent in human cells and *M063R* does not possess a broad-spectrum host range function [15,33]. In our study, the control Δ*M063R* infection similar to the wildtype virus did not cause upregulation of CXCL10 in human CD14^+^ monocytes (**Fig 2C**). Infection by Δ*M062R* in THP1-differentiated macrophages caused significantly higher levels of 2’3’-cGAMP than those from mock treated or cells infected by the wildtype MYXV (**Fig 2E**).

### Infection by Δ*M062R* stimulated IRF-dependent gene expression is sensed through cGAS

**Fig 3:**
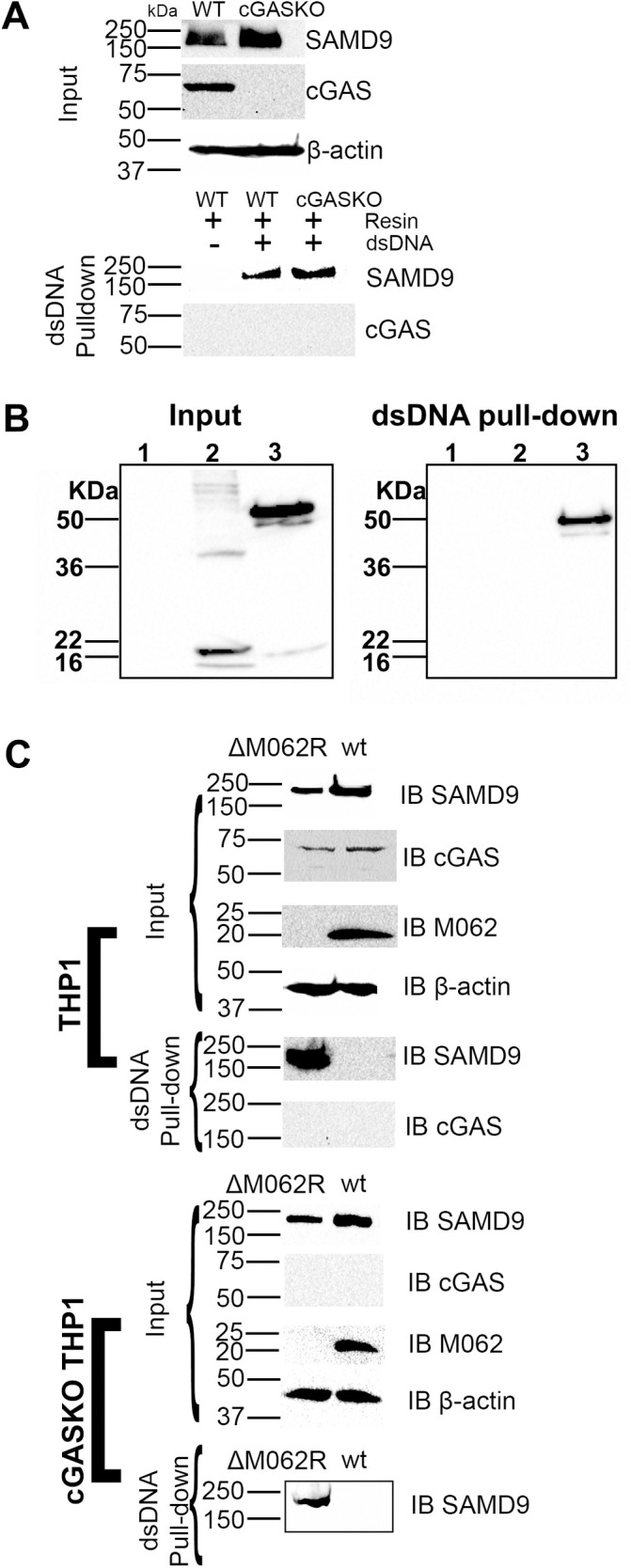
Viral M062 prevents human SAMD9 from being associated with dsDNA in a cGAS independent manner. **A.** DNA pull-down assay shows SAMD9 associated with dsDNA. Cell lysates from differentiated wiltype or cGAS-knockout (cGASKO) THP1 cells are incubated with 5’ biotinylated dsDNA, HSV60mer (35), and after extensive washing bound proteins are eluted for western blot detection of SAMD9 and/or cGAS. **B.** SAMD9 1–385 aa domain but not the N-terminal 1–110 aa is associated with dsDNA. The 5’-biotinylated VACV 70mer dsDNA pull-down experiment as in “A” is performed. Cell lysate from mock transfected, 1–110 aa construct (FLAG tagged) transfected, or 1–385 aa (FLAG tagged) construct transfected cells were used to incubate with dsDNA (VACV 70mer) conjugated resin and the co-precipitated content was separated on SDS-PAGE and probed for FLAG by western blot. Lane 1: mock treated; 2: transfection of 1–110 aa SAMD9; 3: transfection of 1–385 aa SAMD9. **C.** The presence of M062 inhibits the association of SAMD9 with dsDNA. With calf thymus dsDNA cellulose for dsDNA pulldown, we used THP1 cells with cGAS or cGASKO expressing endogenous SAMD9 and infected them with either wildtype MYXV expressing M062 protein or ΔM*062R* MYXV. Proteins associated with DNA were separated on SDS-PAGE for western blot probing for SAMD9 and cGAS.

We have reported previously that the infection defect by Δ*M062R* was due to its inability to overcome host SAMD9 function [27]. However, the immunological impact of Δ*M062R* remains unknown. A computational analysis of SAMD9 across all homologues found putative DNA binding domains [34], and DNA pulldown experiments using either the VACV 70mer dsDNA [35] (**S2 Fig**) or calf thymus dsDNA (**Fig 3A**) indicated that SAMD9 was co-immunoprecipitated with dsDNA in a cell type and dsDNA sequence independent manner. We also tested other dsDNA such as HSV60mer [35] and observed the same ability of SAMD9 in association with dsDNA. We found previously that the MYXV M062 protein binds to amino acids (aa) 1–385 of human SAMD9 but not to the first 285 aa residues or c-terminal portion of SAMD9 [32], while the region of 285–385 aa in human SAMD9 overlaps with the putative DNA binding domain, the Alba-2 domain [34]. We next examined if a human SAMD9 1–385 aa fragment could also be associated with dsDNA. We used the *SAMD9*-null HeLa cells we previously engineered [32] for the experiment and by transiently transfecting the cells to express SAMD9 1–385 aa we performed the dsDNA pulldown experiment similar to that shown in the S2 Fig. We found that the SAMD9 1–385 aa truncated protein also associated with dsDNA **(Fig 3B)**. As a control, we transiently expressed a SAMD9 N-terminal fragment of 1–110 aa that contains the SAM domain in *SAMD9*-null cells, and found that this SAMD9 fragment was not associated with DNA **(Fig 3B)**. We then investigated whether the expression of MYXV M062 might interfere with SAMD9’s presence in the dsDNA pulldown content. As a control, we infected THP1-differentiated macrophages (**Fig 3C**) or HeLa cells (**S3 Fig**) expressing intact endogenous SAMD9 with either wildtype MYXV or Δ*M062R*. In either case, wildtype MYXV infection significantly reduced the amount of SAMD9 associated with dsDNA compared with that from Δ*M062R* infection (**Figs 3C** and **S3**).

**Fig 4:**
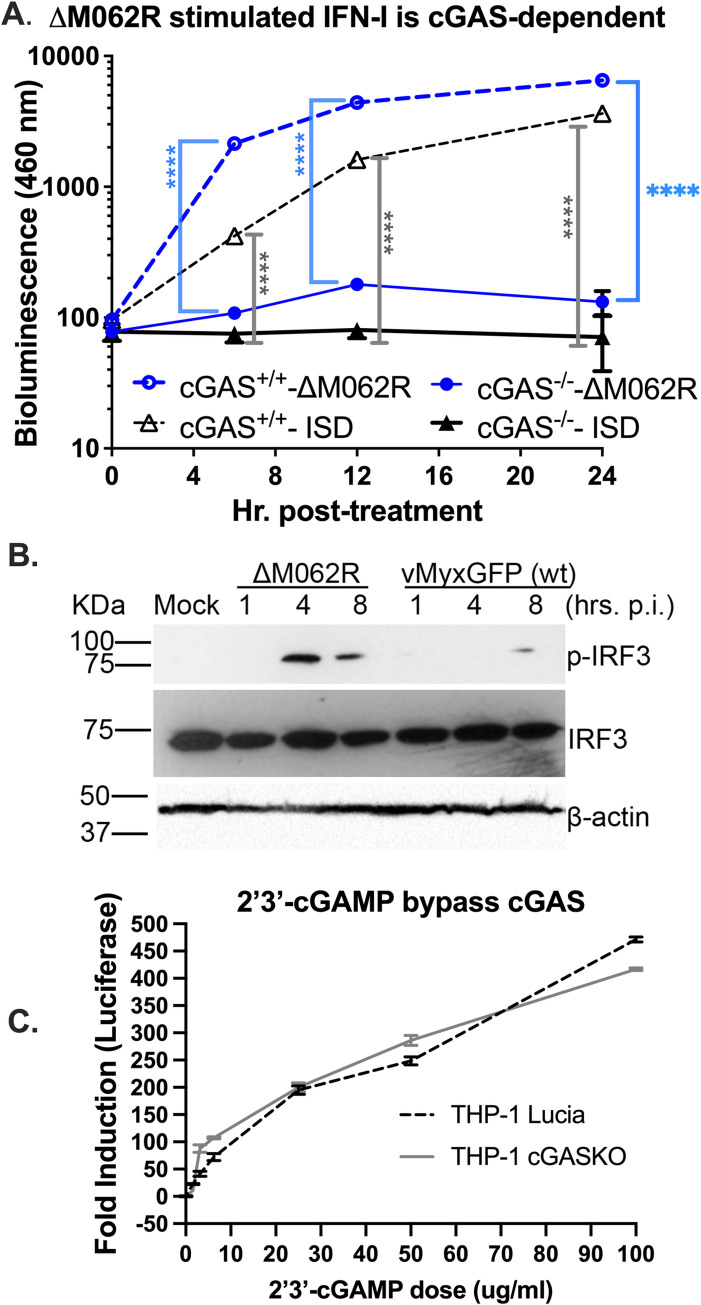
IRF-dependent gene expression stimulated by Δ*M062R* MYXV is regulated through cGAS. **A.** Infection by ΔM*062R* MYXV stimulates similar IRF-dependent gene expression to dsDNA-stimulated effect and is cGAS- dependent. THP-1-Lucia human macrophages with or without cGAS expression were treated by transfection with interferon-stimulated dsDNA (ISDs) or by infection with Δ*M062R* MYXV (at a moi of 10) at given time points for harvesting. Cell supernatant is used for luciferase assay. Shown is the representative of two independent biological replicates and at each given time point the data point is the average of the triplicate (technical replicate). Only the THP-1 cells with intact cGAS were able to respond with increased production of luciferase in either treatment. Two-way ANOVA and multiple comparisons are performed to compare cGAS effect to the luciferase detection with time for either ΔM*062R* or ISD stimulation, and a statistical significance is defined as *p<0.05 and ****p<0.0001. Both time and the presence of cGAS significantly affected the readout (p<0.0001). **B.** Infection by ΔM*062R* MYXV leads to phosphorylation of IRF3. Differentiated THP1 cells are infected with either ΔM*062R* or WT MYXV at a moi of 5 and cell lysates are harvested at the given time points for western blot. A total protein of 25 μg is loaded per sample. Phosphorylated and total IRF3 are probed with β-actin as internal loading control. **C.** Addition of 2’3’-cGAMP bypasses the cGAS deficit and leads to the induction of luciferase activity through the luciferase assay. Transfection of 2’3’-cGAMP at the given doses, the enzymatic product from activated cGAS, produces an identical response in both wild type and cGAS-null THP-1 macrophages, demonstrating that the remainder of the DNA-stimulated IFN-I pathway remains intact. Fold induction at given time point is calculated by normalizing the results to that at the 0 time point. Shown is the average of duplicate (technical replicate) from samples at the given time point and a representative from two independent experiments.

Considering that SAMD9 may function through forming a complex with factors binding to DNA [34], we decided to test whether the ability of Δ*M062R* to induce IFN-I is due to the activation of DNA sensors. We utilized a luciferase expression system in human monocytic THP-1 cells for the study. THP-1 cells can be differentiated into macrophages for testing DNA sensing and downstream outcome, and the firefly luciferase (F-Luc) expression is driven by the IRF recognition domain (Invivogen, San Diego, CA) [28]. To test whether DNA sensing plays a role in Δ*M062R*-induced IFN-I induction and pro-inflammatory responses, we used cGAS-null THP-1 cells that were engineered from the F-Luc expressing parental cells described above [28]. The absence of cGAS in THP1 cells did not affect the ability of SAMD9 to bind to dsDNA (**Fig 2A**). The presence of M062 expression from the wildtype MYXV prevented SAMD9 from binding to dsDNA in a cGAS-independent manner (**Fig 3C**). We found that Δ*M062R* mutant virus stimulated robust luciferase expression comparable to that induced by interferon-stimulating DNA (ISD) [35] transfection (**Fig 4A**). In the absence of cGAS, the luciferase expression caused by both Δ*M062R* and ISD was eliminated (**Fig 4A**). We next confirmed the phosphorylation of IRF3 during Δ*M062R* infection that was detected as early as 4 hours (hrs) post-infection (p.i.) and persisted at 8 hrs p.i.. The wildtype MYXV infection showed minimal level of IRF3 phosphorylation at 8 hrs p.i. (**Fig 4B**) that is most likely mediated through RIG-I dependent RNA sensing [24]. At 16 hrs p.i., Δ*M062R* infection continues to stimulate the phosphorylation of IRF3 to higher levels than that by the wildtype MYXV (**S4 Fig**). However, transfection of 2’3’-cGAMP, the messenger molecule generated by cGAS upon DNA binding, successfully bypassed the lack of cGAS in cGAS-null THP-1 to restore F-Luc expression (**Fig 4C**). We thus conclude that the immunostimulatory effect of Δ*M062R*, distinct from the wildtype MYXV, is due to the activation of the cGAS-dependent DNA sensing pathway.

### Knocking down SAMD9 expression in monocytes/macrophages attenuated their proinflammatory responses

**Fig 5:**
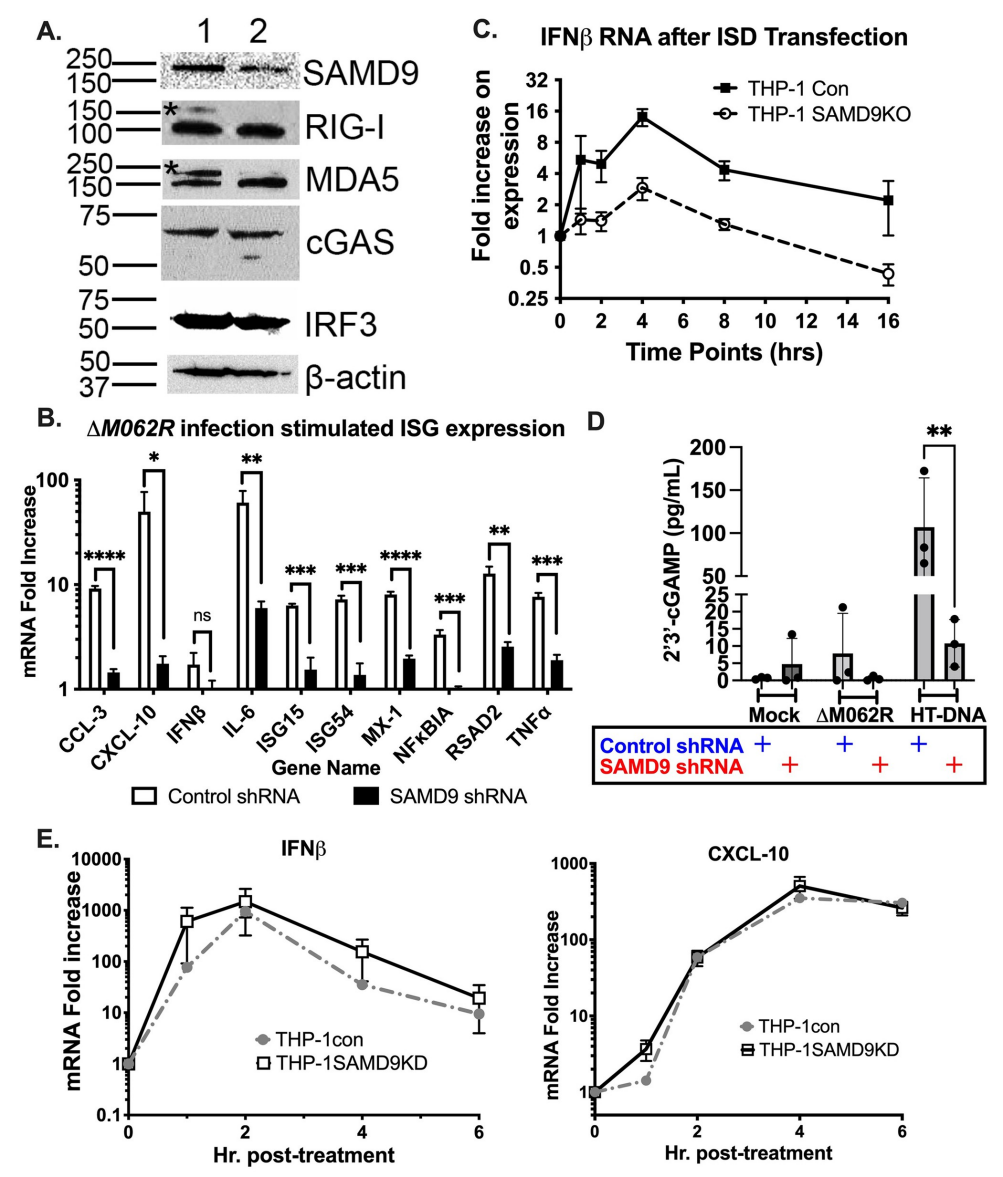
Knocking down SAMD9 expression attenuates the proinflammatory responses in THP-1 cells induced by Δ*M062R* infection and dsDNA. **A.** THP-1 cells in which SAMD9 was knocked down by shRNA. A total protein of 25 μg per cell line is loaded per sample. Western blotting shows reduced SAMD9 expression in THP-1 cells stably-transduced with lentivirus expressing SAMD9 shRNAs. Additional targets such as sensors RIG-I, MDA5, cGAS, and transcription factor IRF3 are also probed to show no significant difference in protein expression in SAMD9 knockdown cells with β-actin as internal loading control. The asterisks in RIG-I and MDA5 blots show the relic of SAMD9 probing due to the primary antibodies used being from the same species and the previous stripping was not effective to rid of anti-SAMD9 antibodies from the blot. **B**. Knocking down SAMD9 expression attenuated the proinflammatory response induced by Δ*M062R*. Compared to control shRNA expressing THP-1, the SAMD9-knockdown cells showed reduced proinflammatory responses after Δ*M062R* infection using RT-PCR. Shown is a representative of 2 independent experiments and each data point is the average of 3 replicates. **C**. ISD-stimulated IFNβ mRNA is reduced in SAMD9 knockdown cells. After ISD transfection, IFNβ mRNA is measured by RT^2^-PCR and in SAMD9-knockdown cells the expression level of IFNβ is reduced. Presented is the representative of two independent experiments and each data point is the average of triplicate in readout. **D**. Knocking down SAMD9 significantly reduces dsDNA induced 2’3’-cGAMP levels in differentiated THP1 cells. Differentiated control and SAMD9-knockdown THP1 cells are mock treated, infected with ΔM*062R* at a moi of 5, or transfected with HT-DNA for 8 hours before cells are harvested for 2’3’-cGAMP ELISA. Shown is the representative of 2 independent experiments and the average of triplicate of technical replicates. Two-way ANOVA followed by multiple comparisons are performed with p<0.05 defined as being statistically significant. **p<0.01. **E.** Transfection of 50 μg/mL of 2’3’-cGAMP bypasses the block on dsDNA-induced proinflammatory responses in SAMD9-knockdown cells. Transfection of 2’3’-cGAMP in both differentiated control and SAMD9-knockdown THP1 cells is performed and cells are harvested at the given time points. RNA extraction followed by RT-PCR is conducted. Both cell lines show comparable kinetics and levels of IFNβ and CXCL-10 mRNA at each time point. Shown is representative data from two independent experiments.

MYXV M062 inhibits SAMD9 function, leading to a productive viral infection. We next examined whether SAMD9 played a role in regulating the proinflammatory responses induced by Δ*M062R*. We generated stable SAMD9 knock-down THP-1 cells using lentivirus expressing shRNAs targeting human SAMD9. As the control, we engineered THP-1 cells stably expressing scrambled shRNAs. The resulting SAMD9-knockdown THP-1 showed partial reduction of SAMD9 protein level while these cells retained expression of RIG-I, MDA5, cGAS, and IRF3 at levels comparable to the control THP-1 cells (**Fig 5A**). We infected differentiated THP-1 control or SAMD9 knockdown cells with Δ*M062R* for 18 hrs before examining pro-inflammatory cytokine production via RT-PCR. We found that reduced SAMD9 expression indeed attenuated Δ*M062R*-induced pro-inflammatory responses (**Fig 5B**). Transfection of ISD into SAMD9-knockdown THP-1 cells showed a similar attenuation in the IFNβ mRNA levels (**Fig 5C**) that is also reflected in the levels of 2’3’-cGAMP (**Fig 5D**). Although the differences in 2’3’-cGAMP levels detected in control and SAMD9-knockdown THP-1 cells infected with Δ*M062R* did not reach a statistical significance, a trend of reduced 2’3’-cGAMP can be seen (**Fig 5D**). However, transfection of 2’3’-cGAMP led to upregulation of the IFNβ and ISG expression in SAMD9-knockdown THP-1 cells (**Fig 5E**) that is similar to the response in the control THP-1 cells. We thus concluded that Δ*M062R* infection stimulated a unique pro-inflammatory state that is cGAS-dependent and also regulated by SAMD9. This effect caused by SAMD9 is not through upregulating sensor or transcription factor protein synthesis (**Fig 5A** showing comparable protein levels of sensors and transcription factor IRF3 between the control and SAMD9 knockdown cells), but is likely to be upstream of 2’3’-cGAMP, which is a secondary message to trigger IFN-I induction.

### Next generation sequencing validated the proinflammatory responses triggered by Δ*M062R* infection and also revealed a unique transcriptomic landscape

**Fig 6:**
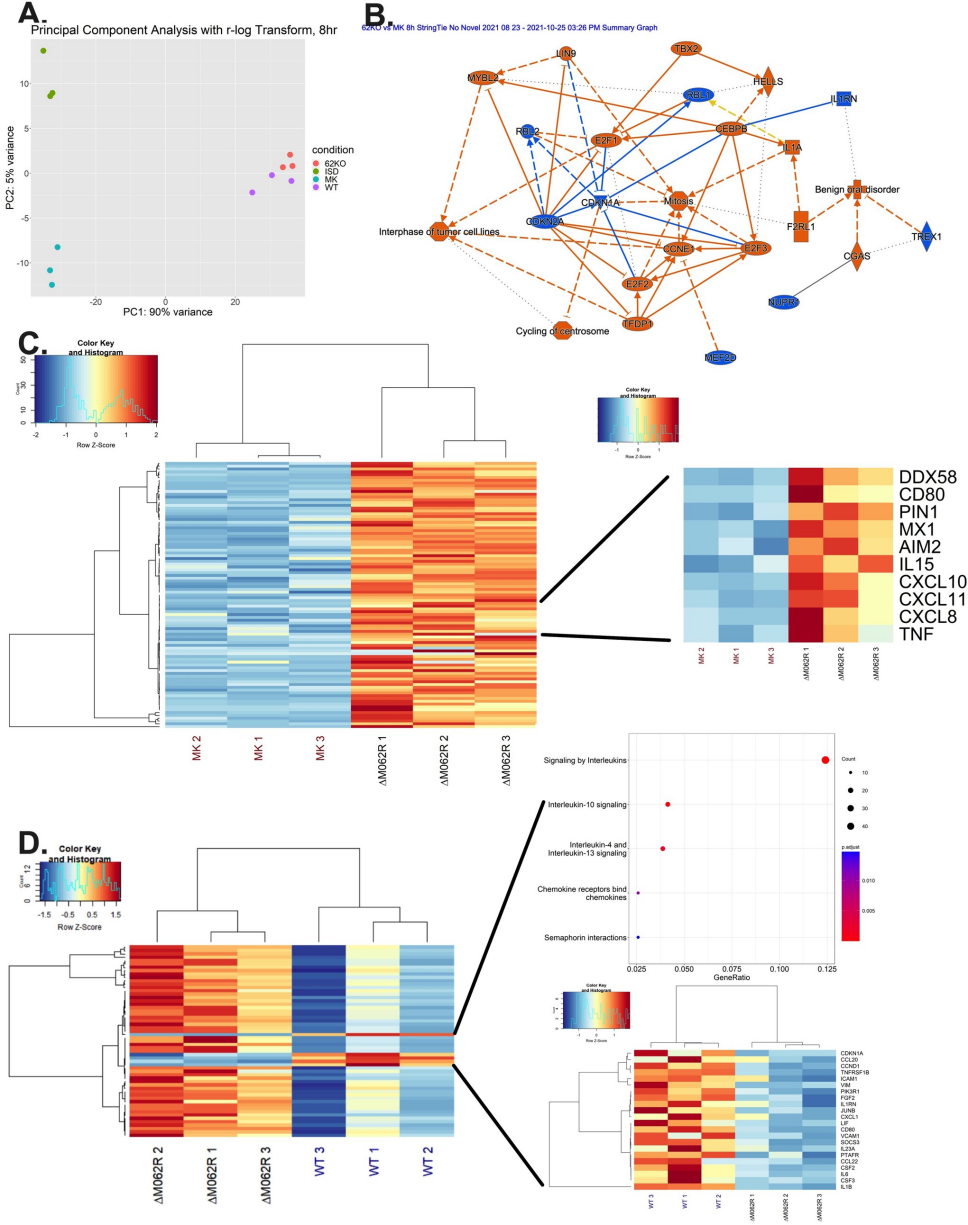
Dual RNAseq analyses reveal a unique host transcriptional profile stimulated by Δ*M062R* MYXV infection that is distinct from that caused by dsDNA alone. **A.** PCA plot of mock, ISD, ΔM*062R*, and wildtype MYXV treated samples. THP-1 cells are differentiated into macrophage-like cells followed by mock treatment, ISD transfection, infection with ΔM*062R* or wildtype MYXV at a moi of 10 for 8 hours before harvesting for RNA extraction, library generation, and next generation sequencing. Triplicate per group (biological replicate) is included for this experiment. Good separation among all four samples are observed with close clustering within replicates. **B.** Ingenuity pathway analysis (IPA) revealed signaling pathway stimulated by Δ*M062R* infection. Host genes differentially expressed during Δ*M062R* infection at 8h post-infection are analyzed using IPA (Qiagen, version 01-20-04) for pathways affected. The graphic summary is shown. The orange color highlighted pathways are upregulated (e.g., cGAS pathway), while pathways in blue are being downregulated. **C.** Heatmap showing distinct host ISG gene expression profile stimulated by Δ*M062R*. All significant differentially expressed genes under study were visualized using the R library heatmap.2. Gene counts were normalized using DESeQ2’s default normalization. Values in each row were scaled using a z-score method before plotting and ward. D hierarchical clustering was performed using the Euclidean distance measure (on the larger heatmap) and no clustering was performed on the break-out heatmap. Representative genes from RT^2^-PCR assay from Fig 2A are shown. **D.** Distinct host transcription profiles between ΔM*062R* and wildtype MYXV infection. All significant differentially expressed genes, between the groups under study, were visualized using the R library heatmap.2. Gene counts were normalized using DESeQ2’s default normalization. Values of each row were scaled using a z-score method before plotting and ward. D hierarchical clustering was performed using the Euclidean distance measure on the larger heatmap, and no clustering was performed on the breakout heatmap. The R library ReactomePA was utilized to perform a pathway enrichment analysis. All downregulated DEGs were sent to the library, and the top 5 enriched pathways are displayed (q-value/p.adjust < 0.1). GeneRatio indicates the ratio between the number of overlapping DEGs in the given pathway, and the total number of DEGs. Further, the size of each dot represents the Count, or the total number of overlapping DEGs in a given pathway.

We conducted a next generation sequencing study in the macrophage-like THP-1 cells to investigate the global transcriptomic change caused by Δ*M062R*. We chose a time point of 8 hours post-infection to perform the study due to the fact that at this time point in comparison to wildtype MYXV, Δ*M062R* infection leads to comparable early gene expression and may be the optimal time to see the effect directly caused by the lack of viral M062 (**Fig 1D**). We hypothesized that since Δ*M062R* infection of macrophages stimulated a cGAS-dependent IFN-I response, the infection will lead to similar results as dsDNA stimulated changes in the transcriptomic landscape. As a control, we included the cells transfected with ISD dsDNA. As a quality control, our data shows distinct separation among 4 groups and clustering in the triplicate within each group (**Fig 6A**), and in Δ*M062R* treated samples the lack of M062R detection is confirmed (**S7 Fig**). Using the dual RNAseq bioinformatic analyses, we found that Δ*M062R* infection in differentiated THP-1 cells stimulated proinflammatory state with activation of the cGAS pathway (**Fig 6B**). While we confirmed the activation of the cGAS pathway by Δ*M062R*, we also confirmed our earlier observation that Δ*M062R* infection led to apparent upregulation of ISGs in comparison to wildtype MYXV infection (**Fig 6C**). Infection by Δ*M062R* leads to the upregulation in the same group of antiviral and proinflammatory factors we identified using the RT^2^-PCR array (**Fig 2A** and **2B**) and ELISA (**Fig 2C**). This is consistent with the fact that in Δ*M062R* infected cells common differentially expressed genes are also detected in ISD treated cells (**S6A Fig**). Unexpectedly, we observed several unique features also induced by Δ*M062R* infection in these macrophage-like cells (**S6 Fig**). First of all, compared to the wildtype MYXV infection-triggered response, Δ*M062R* infection seems to specifically downregulate the immunosuppressive pathways including IL10 and IL4/IL13 pathways, as revealed by both REACTOME and GO analysis (**Fig 6D**). Activation of these cytokine/chemokine pathways is common to the induction of IFN-I responses as it is a part of the feedback mechanism to regulate the duration and intensity of IFN-I responses. In human macrophages, wildtype MYXV infection triggers IFN-I and TNF through the activation of RIG-I [24], which may explain the upregulation of a subgroup of cytokine and chemokine genes by wildtype MYXV that are common to ISD treated samples (**S6A Fig**). We observed phosphorylation of IRF3 in wildtype MYXV infection of macrophage-like cells (**Fig 4B**) likely due to the RIG-I-associated mechanism as previously reported [24], albeit at a much lower level than that stimulated by Δ*M062R* infection (**Fig 4B**). We used flow cytometry to examine the status of phosphorylated IRF3 in wildtype and Δ*M062R* infected cells. We found in PMA differentiated THP1 cells, which is considered to be M0 phenotype and is prone towards proinflammatory commitment, wildtype MYXV infection still suppressed overall levels of phosphorylated IRF3 at this time, while Δ*M062R* infection continued to show increased phosphorylated IRF3 (**S4 Fig**). Another surprising and unique feature of Δ*M062R* infection is the upregulation of genes for cell cycle checkpoints, DNA repair, and processing of capped intron-containing pre-mRNAs, etc, when we compare Δ*M062R* infection with ISD stimulated effect (**S5B Fig**). Such a unique patterns of gene regulation may have a positive impact to somatic diversification of the immune receptors, nucleic acid metabolic process, and possibly survival, to name just a few biology processes identified from Gene Ontology (GO) enrichment analysis (**S5A Fig**).

We also examined the viral gene expression (transcription) profile of Δ*M062R*, as the Δ*M062R* MYXV is not yet evaluated for the viral transcriptional status. The time point choice of 8 hours post-infection provides a snapshot of viral RNA levels in human macrophages with the lack of M062 in Δ*M062R* infection not causing an apparent effect to other early viral protein synthesis in comparison to the wildtype MYXV infection. Surprisingly, in the Δ*M062R* infection we detected all the viral gene RNAs expressed in the wildtype MYXV infection (**S6B Fig**) including all the post-replicative genes. We found 57 viral genes in Δ*M062R* infection were differentially expressed compared to wildtype MYXV with statistical significance (p value ranging from 1.03e-03 to 2.72e-88). Most of them (52 viral genes) (**Table 1**) showed slightly higher levels in Δ*M062R* infected cells than that in wildtype MYXV infection. For instance, *M136R* expression in Δ*M062R* infected cells is 2.6-fold (logFC = 1.4) higher than that expressed in wildtype MYXV infection (**Table 1**). Only 5 viral genes, *M062R*, *M102L*, *M103L*, *M106L*, and *M107L*, showed significant reduction at RNA levels during Δ*M062R* infection compared to that in wildtype MYXV infection. Among these viral genes, only *M062R* transcript was noticeably reduced (logFC = -3.95) due to the deletion of the central 80% of the *M062R* coding sequence [26] (**S7 Fig**) and the reduction in the RNA levels of the remaining 4 viral genes are in the range of 0.44–0.64 (logFC between -1.16 and -0.652) (**Table 1**).

**Table 1:** Differentially expressed viral gene in Δ*M062R* and wildtype MYXV in human macrophages.

Uniprot ID	Gene ID	Expression kinetics	logFC	p-Value	Conservation within poxviruses
MYXV_gp005	M003.2L	Unknown	0.8	1.34e-03	Semi-conserved
MYXV_gp007	M004.1L	Late	0.801	3.22e-03	Unique
MYXV_gp008	M005L (M-T5)	Early	0.835	9.9e-05	Semi-conserved
MYXV_gp009	M006L	Early	0.928	6.18e-6	Semi-conserved
MYXV_gp011	M008L	Early	0.6980	7.88e-05	Semi-conserved
MYXV_gp012	M008.1L	Late	0.901	8.94e-06	Unique
MYXV_gp015	M011L	Early	0.637	8.45e-04	Semi-conserved
MYXV_gp017	M013L	Early	0.489	4.18e-03	Unique
MYXV_gp022	M018L	Early	0.544	4.39e-03	Semi-conserved
MYXV_gp023	M019L	Late	0.59	3.57e-03	Conserved VACV F9L
MYXV_gp028	M024L	Early	0.883	4.32e-06	Semi-conserved
MYXV_gp031	M027L	Late	0.93	1.97e-06	Conserved VACV E1L
MYXV_gp032	M028L	Late	0.617	4.87e-04	Conserved VACV E2L
MYXV_gp035	M031R	Early	0.429	1.77e-03	Semi-conserved
MYXV_gp036	M032R	Late	0.604	1.78e-04	Conserved
MYXV_gp038	M034L	Early	0.67	3.94e-04	Conserved
MYXV_gp053	M049R	Early	0.39	5.72e-03	Semi-conserved
MYXV_gp055	M051R	Unknown	0.54	1.18e-03	Semi-conserved VACVG6R
MYXV_gp057	M053R	Intermediate	0.746	1.27e-04	Conserved VACV G8R
MYXV_gp058	M054R	Late	0.824	2.44e-05	Semi-conserved VACV G9R
MYXV_gp059	M055R	Late	0.643	5.12e-04	Conserved VACV L1R
MYXV_gp062	M058R	Late	0.507	1.77e-03	Conserved VACV L4R
MYXV_gp066	M062R	Early/Late	-3.95	2.72e-88	Conserved VACV C7L
MYXV_gp067	M063R	Early/Late	0.56	5.56e-04	Semi-conserved VACV C7L
MYXV_gp072	M068R	Early or Intermediate	0.416	5.82e-03	Conserved VACV J6R
MYXV_gp077	M073R	Early	0.6450	2.36e-04	Semi-conserved VACV H5R
MYXV_gp078	M074R	Early/Late	0.8280	1.5e-05	Conserved VACV H6R
MYXV_gp079	M075R	Early	0.505	2.3e-03	Semi-conserved VACV H7R
MYXV_gp088	M084R	Early	0.774	3.37e-05	Conserved VACV D9R
MYXV_gp106	M102L	Late	-0.8610	4.51e-04	Semi-conserved VACV A13L
MYXV_gp107	M103L	Late	-1.16	3.54e-05	Conserved VACV A14L
MYXV_gp110	M106L	Late	-0.6520	4.81e-04	Conserved VACV A16L
MYXV_gp111	M107L	Late	-0.8240	1.57e-03	Semi-conserved VACV A17L
MYXV_gp112	M108R	Intermediate	0.969	6.44e-06	Conserved VACV A18R
MYXV_gp114	M110L	Late	0.802	9.72e-04	Conserved VACV A21L
MYXV_gp119	M115L	Late	0.918	1.23e-06	Semi-conserved VACV A27L
MYXV_gp120	M116L	Late	1.02	2.7e-07	Conserved VACV A28L
MYXV_gp121	M117L	Early	0.535	1.11e-03	Conserved VACV A29L
MYXV_gp122	M118L	Late	0.678	2.5e-04	Conserved VACV A30L
MYXV_gp123	M119L	Early	0.582	1.38e-03	Unique
MYXV_gp126	M122R	Late	0.888	1.02e-04	Semi-conserved VACV A34R
MYXV_gp133	M129R	Early	0.615	1.37e-03	Semi-conserved VACV E7R
MYXV_gp134	M130R	Early	0.515	9.06e-04	Unique
MYXV_gp140	M136R	Late?	1.4	5.54e-11	Unique VACV A52R
MYXV_gp144	M140R	Early	0.599	1.06e-03	Semi-conserved VACV A55R
MYXV_gp145	M141R	Early	0.734	2.93e-04	Unique
MYXV_gp148	M144R	Early	0.463	4.99e-03	Semi-conserved VACV N1L
MYXV_gp153	M150R	Early	0.565	1.03e-03	Unique VACV C9L
MYXV_gp154	M151R	Early	0.792	9.40e-05	Semi-conserved VACV SPI-2
MYXV_gp155	M152R	Early	0.843	5.60e-05	Unique
MYXV_gp158	M156R	Early	0.638	2.51e-04	Semi-conserved VACV K3L
MYXV_gp159	M008.1L	Late	0.893	9.81e-06	Unique
MYXV_gp160	M008L	Early	0.694	8.45e-05	Semi-conserved
MYXV_gp162	M006R	Early	0.931	5.51e-06	Semi-conserved
MYXV_gp163	M005R (M-T5)	Early/Late	0.833	1.05e-04	Semi-conserved
MYXV_gp164	M004.1L	Late	0.791	3.37e-03	Unique
MYXV_gp166	M003.2L	Unknown	0.787	1.45e-03	Semi-conserved

## Discussion

Poxviruses are large dsDNA viruses with an exclusively cytoplasmic life cycle. These viruses can effectively inhibit IFN-I induction through many strategies. These strategies include blocking IFN-I signaling through decoy receptors [36], inhibiting key signaling effector molecules of the sensing pathways [6,8], and/or reprograming host gene expression in the nucleus [19,37,38]. One critical ability is for mammalian poxviruses, especially virulent poxviruses, to circumvent host immunosurveillance and the antiviral immune responses induced as a result of DNA sensing [6]. Myxoma virus belongs to the genus of *Leporipoxvirus* and has a narrow host tropism, causing infectious disease only in rabbits. In European rabbits, virulent MYXV causes diseases with 100% lethality and profound immunosuppression [39]. Despite its limited host tropism for infectious diseases, we found wildtype MYXV inhibits the DNA sensing pathway in human cells comparably to VACV, suggesting the presence of an antagonistic mechanism directed against host DNA sensing in a species-independent manner.

Host detection of cytoplasmic DNA by the cyclic GMP-AMP synthase (cGAS) leads to the production of the second messenger molecule 2’3’-cGAMP; binding of 2’3’-cGAMP to the adaptor, the stimulator of interferon genes (STING), triggers the activation of IFN-I production through a series of signaling events involving STING activation, recruitment and activation of TBK1, and phosphorylation of IRF3 [40]. Many poxvirus proteins inhibit DNA sensing and IFN-I induction through different mechanisms, such as direct degradation of 2’, 3’-cGAMP by Poxins [9,10], inactivation of STING through mTOR by VACV F17 [12], inhibition of IRF3 by VACV C6 [41], and inhibition of NF-κB activation by many VACV proteins including B14 [42] and F14 [43], etc. More importantly, inhibition of host DNA sensing by poxviruses during early infection seems to be a common strategy in spite of their species tropism. Poxvirus early and post-replicative gene expression can be distinguished through the use of a DNA replication inhibitor such as AraC. It is not surprising that poxviruses from different genera diverge on strategies to evade DNA sensing with distinct mechanisms. In the MYXV genome, homologs of poxins are not found and a homolog of VACV F17 is predicted to be a late protein. MYXV may encode one or multiple additional early genes uniquely functioning as inhibitors of the DNA sensing pathway. In this study, we identified one MYXV viral protein which functions in such a capacity.

The MYXV *M062R* gene belongs to one of most conserved poxvirus host range factor families based on amino acid similarity using common algorithms, the poxvirus *C7L* superfamily [15]. The prevalence of the *C7L* superfamily homologs is in stark contrast to two other classic orthopoxvirus host range factors, CP77 and K1 [44,45], whose homologs are often fragmented or disrupted even within the orthopoxvirus genus [46]. Among all sequenced mammalian poxviruses, there are some exceptions who do not possess homologs of the *C7L* superfamily, including parapoxviruses, molluscipoxvirus (Molluscum contagiosum virus), squirrel poxvirus, and pteropoxvirus, etc [47,48]. Such prevalence of the *C7L* superfamily among mammalian poxviruses brought the interesting question on their potential overlapping functionality in the host cells. In MYXV, M062 protein is a host tropism determinant of MYXV and essential for viral replication [26]. Although MYXV *M062R* can compensate for the function of the VACV *C7L* gene [49], it is predicted to have functional divergency from C7 and other *C7L* family members of orthopoxviruses [15]. The MYXV M062 is unique from C7 in that [a] in VACV the *C7L* gene is non-essential and [b] only when *C7L* and another orthopoxvirus host range gene *K1L* are both deleted will the defect in host range tropism (comparable to Δ*M062R* MYXV) and replication deficiency become apparent [44]. Our lab found that VACV C7 has a distinct host target from MYXV M062, which finding is also supported by others [50]. A known function of the MYXV M062 protein is to inhibit the function of the host protein SAMD9 [26,27], but the direct immunological impact of the MYXV M062 protein is not known. More importantly, the immunotherapeutic potential of *M062R*-null MYXV as an adjuvant for cancer therapy [14] further motivated our investigation of its immunostimulatory mechanism. Targeted deletion of *M062R* gene in the MYXV genome resulted in a mutant virus that maintained early gene expression at the protein levels but showed reduced DNA replication without late viral proteins being detected through western blot [26]. Interestingly, in our RNAseq analyses we not only detected post-replicative viral RNA, especially late viral RNAs during Δ*M062R* infection, but also found Δ*M062R* viral RNA synthesis patterns in macrophages closely resembling that of the wildtype MYXV infection. We observed a moderate reduction in RNA levels among only 4 late genes during Δ*M062R* infection, and most of the Δ*M062R* viral transcripts were present at slightly higher levels than that in wildtype MYXV infection at the same time point. The presence of significant levels of late RNA during Δ*M062R* infection is unexpected, as in order to synthesize late viral RNA comparable to the wild type virus level, sufficient intermediate proteins must be produced *de novo*. This phenotype suggests a unique antiviral state stimulated by Δ*M062R* MYXV infection. In this state, viral protein synthesis, especially late protein production, is inhibited, which is coupled with inhibition of viral DNA replication. We speculate that the unique antiviral effect of inhibiting viral protein synthesis may be connected to the DNA sensing event. An alternative possibility is that the absence of M062 during Δ*M062R* infection may lead to translation deceleration of viral proteins until a complete stop, when a large quantity of late viral proteins are needed to complete the life cycle. In this alternative scenario, the DNA-triggered immune response reported may be caused by suboptimal levels of viral immunoregulatory proteins. This is, however, less probable because of seemingly normal viral gene expression at both RNA and protein levels at 8 hours p.i., and the observed robust IRF-dependent gene expression triggered by Δ*M062R*, comparable to what is directly induced by dsDNA as shown in the luciferase assay.

**Fig 7:**
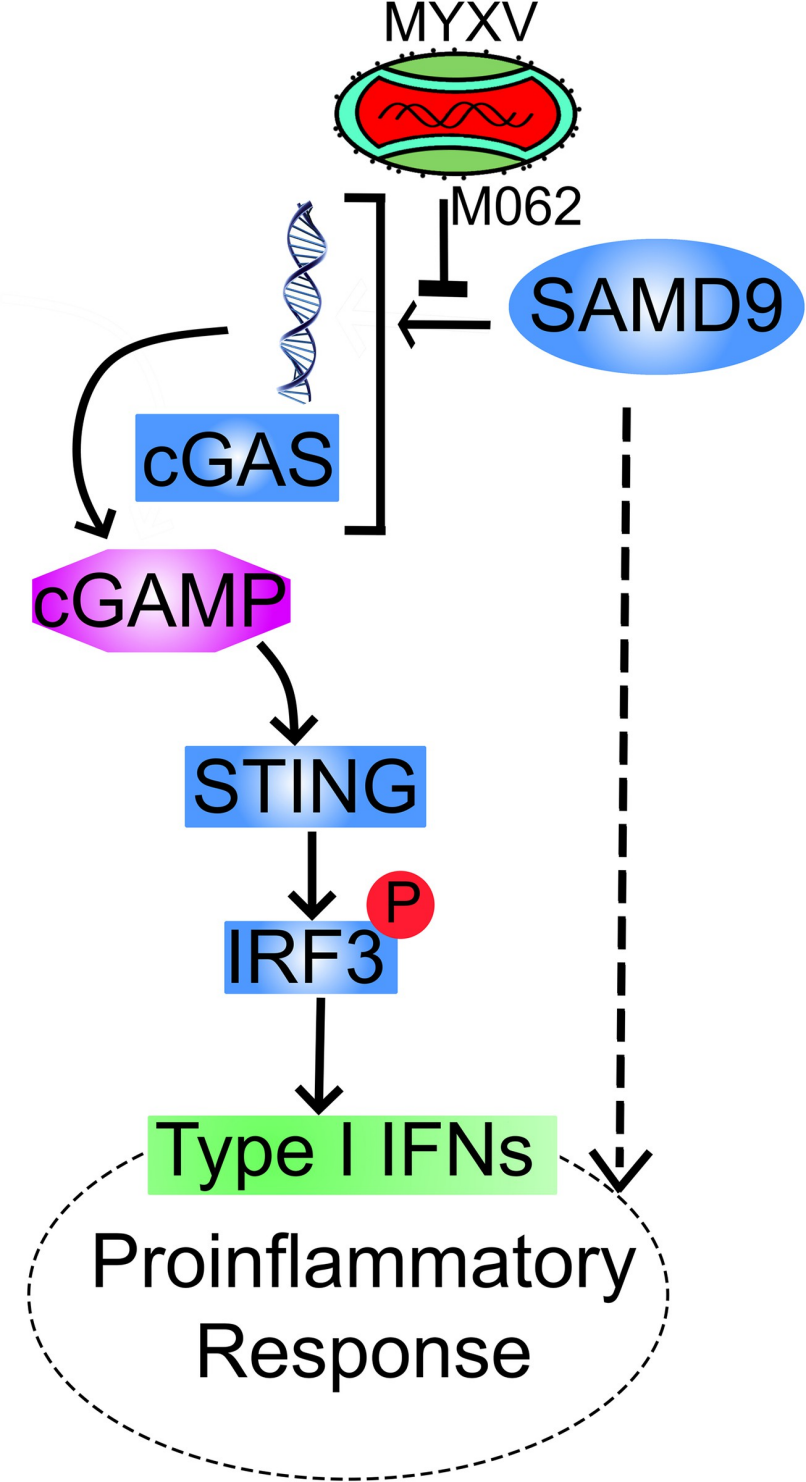
Summary of SAMD9 effect to DNA sensing pathway and proinflammatory responses. Based on our finding, the lack of *M062R* during MYXV infection induces cGAS-dependent IFN-I production that is also regulated by SAMD9. The SAMD9 effect is at the upstream of the 2’3’-cGAMP production but does not significantly affect cGAS protein synthesis. Overall, SAMD9 plays a positive role in enhancing cGAS-dependent IFN-I induction and regulating the proinflammtory responses in our model system (the dotted line).

Cytoplasmic sensing of DNA to trigger protective inflammation plays a key role in host antiviral defense [51,52]. The origins of cytoplasmic DNA may vary during the lifetime of a mammalian cell, such as improperly processed cellular DNA due to DNA repair or replication defect [31], nuclear membrane breakdown in senescent cells [53], mitochondria (mt) membrane rupture leading to release of mtDNA into the cytoplasm [54], and foreign DNA such as during viral infection [6,55]. Once triggered, DNA sensing induced IFN-I production and inflammation will lead to dramatic changes in the immunological milieu that may ultimately alter the immune responses profoundly. Thus, there must be additional control mechanisms to monitor, regulate, and ultimately limit DNA-stimulated inflammation. There are known downstream host control mechanisms to fine tune IFN-I responses. Other than the negative feedback cascade to restrict the duration and extent of IFN-I responses, (e.g., SOCS), regulation of IFN-α receptor (IFNAR), and USP18 [56–58], intracellular signaling events and miRNAs can also perform such function [59]. Upstream of IFN-I production, there are also regulatory measures to pattern recognition receptors (PRRs) and their downstream pathway components, e.g., AKT to cGAS [60], TMEM120A to STING [61], and RNF138 to TBK1 [62]. Our work provides evidence of a novel mechanism to fine tune the IFN-I response, which may operate through additional regulator(s) of PRR activation (**Fig 7**), and ultimately alters the global transcriptional landscape.

SAMD9 is a large and exclusively cytoplasmic protein with a complex domain structure, interestingly including putative DNA-binding domains [34]. Our study indicated that although SAMD9 can be associated with dsDNA in a sequence-independent manner, SAMD9 is not necessarily a direct DNA sensor, since the triggering of IFN-I induction and subsequent proinflammatory responses is through cGAS-dependent DNA sensing events. During wildtype MYXV infection, cGAS may not have access to MYXV genome DNA in the cytoplasm in order to induce IFN-I. In the presence of M062 protein, SAMD9 is located exclusively outside of the cytoplasmic viral factories [27]. However, during Δ*M062R* infection, SAMD9 can be detected abundantly associated with the viral factories [27]. Because of its putative DNA binding domain and its complex domain structure [34], during poxvirus infection SAMD9 may serve as an enabler to facilitate the access to cytoplasmic DNA by sensors such as cGAS.

While this study is under review, a parallel study showed *in vitro* that the relatively small binding domain in SAMD9 where M062 happens to target could also bind to dsRNA [63], we could not confirm such observation using the full-length SAMD9 in the absence of poxvirus infection based on our negative results in co-localizing cellular SAMD9 with dsRNAs including polyI:C many years in the past. Moreover, MYXV also cannot evade human RNA sensing mechanism, such as through RIG-I [24] and Δ*M062R* does not trigger phosphorylation in eIF2alpha that is often trigged by dsRNA activated PKR, we could not further validate the biology of whether wildtype SAMD9 also facilitates dsRNA sensing or if M062 binding to SAMD9 prevents SAMD9 from binding to dsRNA. Finally, in cGASKO THP1 cells, Δ*M062R* stimulated IRF3-dependent gene expression is eliminated, thus M062 direct binding to SAMD9 most likely has a dominate impact to the DNA sensing associated immunological consequences. In this study we found that the levels of 2’3’-cGAMP (**Fig 5D**) and phosphorylation of IRF3 (**Figs 4C** and **S4**) induced by the Δ*M062R* are moderate compared to those caused by direct dsDNA transfection. This suggests that in spite of the presence of large quantity of viral genome DNA in the cytoplasm during Δ*M062R* infection [27] cGAS still may not have full access to these DNA to generate large quantity of 2’3’-cGAMP probably due to the intact architecture of viral factories. Such cGAS access to viral DNA during Δ*M062R* infection is likely rendered by SAMD9 due to its ability of being associated with dsDNA and wrapping around the viral factories [27]. The observation that after Δ*M062R* treatment a new exposure of dsDNA triggered significant increase of IRF-dependent gene expression (**Fig 1B**) is intriguing. This reminds us of an interesting phenomenon reported recently, called trained innate immune memory [64], in which functional reprogramming of innate immune effectors, such as monocytes/macrophages, through an initial exposure to a microbial component leads to reinstating of avid cytokine production during a challenge with another microbial stimulus from the same class. Treatment by Δ*M062R* also leads to reprogramming with an apparent proinflammatory signature (**Fig 6B** and **6C**) but different from that in macrophages with dsDNA transfection alone (**S5 Fig**). Such transcriptional reprogramming by Δ*M062R* promotes capped RNA processing, DNA replication, and proliferation, just to name a few, and can occur at as early as 8 hours post infection. We reason that such effect reflected in the transcriptional regulation is unlikely due to additional extracellular stimuli such as from paracrine/autocrine IFN-I responses, since Δ*M062R* induced IRF3 phosphorylation starts at 4 hours post-infection and persists at 16 hrs p.i., while 2’3’-cGAMP production can be observed at 8 hrs p.i.. Future investigation is needed to further define the effect caused by Δ*M062R* to compare with that during the establishment of the trained innate immune memory and to characterize the immunological consequences in monocytes/macrophages.

SAMD9 participated in the regulation of host translation in the initiation and plays an important role in the elongation steps, and deleterious mutations in SAMD9 can inhibit global protein synthesis [65]. More importantly, SAMD9 may serve as a signaling hub to fine tune the immunological consequences of the cell [34]. SAMD9 may be targeted by other viruses [66] and has broad-spectrum antiviral effect [67–69]. In our pursuit of understanding the mechanism of the SAMD9 antiviral effect, through the use of Δ*M062R* as a tool, we revealed a previously unrecognized role of SAMD9 in regulating innate immune DNA sensing. The question remains that how translation regulation impacts the transcriptomic landscape in our observation. One of the hypotheses is that cytoplasmic remodeling of protein synthesis may have a feedback effect to the nuclear transcription reprograming. While we cannot yet rule out a direct role of SAMD9 to transcription regulation in the nucleus, further investigation will certainly be needed. Lastly, it is now recognized that innate immune signaling machinery may be reprogrammable and have flexibility to generate custom-defined outcomes [70]. Such theory suggests cross-talks among cytoplasmic signaling pathways that may be more intimate than what we currently imagined, a third alternative model. The transcriptional remodeling in monocytes/macrophages we reported here induced by Δ*M062R* and regulated by SAMD9 presents us an invaluable opportunity to understand such intricate machinery and its regulation. This work leads us to investigate its function in connecting innate immune sensing, translation control, and transcriptional refinement in host immune responses as the next step.

## Materials and methods

### Cell culture, plasmid constructs, and virus stock

Mammalian cells used in this study include BSC-40 (ATCC CRL-2761) (in Dulbecco minimal essential medium, DMEM, Lonza), healthy human peripheral CD14^+^ monocytes (Lonza, Walkersville, MD), THP1 (ATCC TIB-202), and THP1-Lucia (kindly provided by F. Zhu) [71] (in RPMI1640, Lonza). The complete growth medium (e.g., DMEM Lonza/BioWhittaker Catalog no 12-604Q, or RMPI1640) was supplemented with 10% FBS (Atlanta Biologicals, Minneapolis, MN), 2 mM glutamine (Corning Cellgro, Millipore Sigma, St. Louis, MO), and 100 μg per ml of Pen/Strep (Corning Cellgro, Millipore Sigma, St. Louis, MO); for RPMI1640 complete culture medium, in addition to FBS, glutamine, and Pen/Strep, 2-mercaptoethanol (MP biomedicals, Solon, OH) was supplemented to a final concentration of 0.05 μM.

Plasmids used in this study are published and described previously [32]. Briefly, SAMD9 fragment 1–110 amino acid (1–110 aa) and 1–385 aa are cloned in the vector of pTriEx-3xFLAG (N-terminal tag) for mammalian expression. After transfection exogenous gene expression is detected by anti-FLAG antibody.

The viruses used were all derived from myxoma virus (MYXV), Lausanne strain (GenBank Accession AF170726.2). The MYXV *M062R* deletion mutant (vMyxM062RKOgfp or Δ*M062R*) and the wildtype MYXV with early/late poxvirus promoter driven GFP expression have been described previously [26]. Vaccinia virus used in this study is WR strain described previously [29]. Virus stocks were prepared on BSC-40 cells and purified with sucrose step gradient through ultracentrifugation as previously described [72].

### RT^2^ Profiler PCR Array, Multi-plex Cytokine Array, and 2’3’-cGAMP ELISA

Primary human CD14^+^ monocytes/macrophages (Lonza, Walkersville, MD) were mock treated, infected with wildtype MYXV and vMyxM062RKOgfp at a multiplicity of infection (moi) of 5 for 16 hours (hrs) before harvesting RNA for RT Realtime (RT^2^) PCR profiler and collecting supernatant for the Multi-plex Array study. For RT^2^ Profiler PCR Array Human Interferons and Receptors (Cat# PAHS064ZC-12, Qiagen, Germantown, MD), RNA extraction (RNeasy Pls Mini kit, Qiagen), cDNA synthesis (RT^2^ First Strand Kit, Qiagen), and Realtime PCR (RT^2^ SYBR Green qPCR Mastermixes, Qiagen) were performed following standard instructions from the manufacturer. The result is representative data from 1 individual and a total of 2 healthy individuals’ CD14^+^ monocytes which were tested (biological replicates). For CXCL10 and IFN α, we used Cytokine Multiplex of Human Inflammation Panel (Invitrogen™ Inflammation 20-Plex Human ProcartaPlex Panel) (Catalog # EXP20012185901, eBioscience/Invitrogen). The procedures were performed following manufacturer protocol and the plate was processed on BioRad Bio-Plex 200 system with Bio-Plex-HTF attachment (Bio-Rad, Hercules, CA). The result is the representative from one of 2 healthy individuals’ CD14^+^ monocytes. Shown is the average intensity from duplicate samples (technical replicates). Detection of 2’3’-cGAMP is performed using 2’3’-cGAMP ELISA Kit (Cayman Chemical, Catalog# 501700) following the standard manufacturer protocol. THP-1 cells are treated with control or viral infection and at 8 hours post-treatment samples are harvested for analysis. Two biological replicates are performed and in each experiment 3 to 4 replicates are used as technical replicate.

### Purification of human peripheral CD14^+^ monocyte/macrophage

Healthy human whole blood was collected by venous puncture into collection tubes (BD catalog# 364606, Vacutainer ACD Solution), approximately 8 mL of whole blood per tube. An equal volume of sterile, room-temperature Dulbecco’s Phosphate Buffered Saline without calcium and magnesium (DPBS) (Corning catalog# 21-031-CV) was added to the flask. The diluted whole blood was layered over Lymphoprep solution (Accurate Chemical and Scientific Corp., Catalog# 1114545) and centrifuged at 2,500 rpm for 20 minutes. The collected PBMCs were 1:1 diluted with DPBS. The tube was then centrifuged at 1500 rpm for 5 minutes and the cell pellet was resuspended in 20 mL DPBS before repeating the wash process once more. The pellet was resuspended in 0.5 mL DPBS with 0.5% BSA and labeled with 50 μL anti-CD14 microbeads (Miltenyi Biotec, catalog# 130-050-201). Cells were labeled for at least 20 minutes at 4°C and CD14^+^ cells were isolated by magnetic column (Miltenyi Biotec, catalog# 130-042-201).

### Luciferase assay

Supernatant from each sample was collected and immediately used in the luciferase assay. Luciferase presence in the supernatant was quantified by kit (QUANTI-luc, InvivoGen, catalog number rep-qlc1). Each sample was tested in triplicate in white 96-well plates with clear bottoms (LUMITRAC 200, Greiner Bio-One, Monroe, NC) with 5 times of volume to the supernatant sample. For each sample in triplicate the arithmetic average was reported. Fold induction is calculated as previously reported [6].

### Semi-quantitative RT Realtime PCR

THP-1 cells (10^6^ cells per 3.5 cm dish) were differentiated in the presence of PMA at 50 ng/mL for 48 hours (hrs) before being mock treated, transfected with ISD [35] at 2 μg per dish using ViaFect (Promega, Madison, WI) at 3 μL per 1 μg of ISD based on manufacturer protocol, or infected at an moi of 5 for either WT (vMyxGFP) or *M062R*-null MYXV (Δ*M062R*) [26]. At 1 hour (h) and 8 hrs post-transfection for ISD or 1 hr and 12 hrs post-infection, cells were harvested with Direct-zol RNA Mini Prep kit (catalog # R2052, Zymo, Irvine, CA) according to manufacturer standard protocol. RNA quality is examined by running on the RNA gel to check 28S and 18S integrity and spectrophotometer to estimate concentration. Equal amount of total RNA in a maximal volume of 6 μL is used for cDNA synthesis using NEB ProtoScript First Strand cDNA Synthesis Kit (Catalog # E6300L, NEB Inc, Ipswitch, MA) as instructed in the manufacturer standard protocol. Realtime PCR is conducted following manufacturer standard protocol (Luna Universal qPCR Master Mix, NEB Inc). Sybr green RT-PCR primers used in this study is listed in **Table 2**.

**Table 2:** Real-time PCR Primer sequences.

Target Gene	Primer sequences
AIM2	Forward (Fwd): 5’- CTCCTGAGTCCTCTGCTAGTTA -3’
	Reverse (Rev): 5’- ACTCTCCATCTGACAACTTTGG -3’
CXCL-10	Fwd 5’- CTG TAC CTG CAT CAG CAT TAG TA -3’
	Rev 5’- GAC ATC TCT TCT CAC CCT TCT TT -3’
CYLD	Fwd 5’- gtgggctgtcctgtgaaagta -3’
	Rev 5’- aagctgtttcccttggtaca -3’
IFIH1	Fwd 5’- accaaatacaggagccatgc -3’
	Rev 5’- gcgatttccttcttttgcag-3’
IFN B1	Fwd 5’- GCC ATC AGT CAC TTA AAC AGC -3’
	Rev 5’- GAA ACT GAA GAT CTC CTA GCC T -3’
IL1B	Fwd 5’- AAGTACCTGAGCTCGCCAGTGAAA -3’
	Rev 5’- TTGCTGTAGTGGTGGTCGGAGATT -3’
ISG15	Fwd 5’- gcgaactcatctttgccagt -3’
	Rev 5’- cttcagctctgacaccgaca -3’
ISG54	Fwd 5’- AGCGAAGGTGTGCTTTGAGA -3’
	Rev 5’- GAGGGTCAATGGCGTTCTGA -3’
MX-1	Fwd 5’- ctcccactccctgaaatctg -3’
	Rev 5’- ttcggaaacaaccatcttcc -3’
NFκB1A	Fwd 5’- CCCTACACCTTGCCTGTGAG -3’
	Rev 5’- TGACATCAGCACCCAAGGAC -3’
RSAD2	Fwd 5’- AGT GCA ACT ACA AAT GCG GC -3’
	Rev 5’- CTT GCC CAG GTA TTC TCC CC -3’
STAT1	Fwd 5’- CTA GTG GAG TGG AAG CGG AG -3’
	Rev 5’- CAC CAC AAA CGA GCT CTG AA -3’
TNFα	Fwd 5’- TCCCCAGGGACCTCTCTCTA -3’
	Rev 5’- GAGGGTTTGCTACAACATGGG -3’

### SAMD9 knockdown and control THP-1 cells

Lentiviral particles for stable SAMD9 shRNA expression (Cat# sc-89746-V, Santa Cruz Biotechnology, Dallas, TX) and Lentiviral particles for stable expression of scrambled shRNAs (control) (Santa Cruz Biotechnology, Dallas, TX) were used for generating the cell lines. We followed the manufacturer’s standard protocol similar to that reported before (26), and stable expression was selected under puromycin at 5 μg/mL. SAMD9 knock-down was confirmed by western blot using rabbit anti-SAMD9 antibody (Cat# HPA021319, Millipore-Sigma, St. Louis, MO) and goat-anti-rabbit HRP secondary antibody (Cat# 111-035-144, Jackson ImmunoResearch Laboratory, INC, West Grove, PA) according to manufacturer’s instructions.

### Transfection

Transfection of plasmid DNA is performed using ViaFect transfection reagent (Promega, Catalog # E4981) based on recommendation of manufacturer’s standard protocol and transfectagro Reduced Serum Medium (Corning, Catalog # 40-300-CV) was used as based medium. Transfection of 2’3’-cGAMP is performed using Lipofectamine RNAiMAX transfection reagent (Invitrogen, Catalog # 13778075) based on the standard protocol provided by the manufacturer. After dose response testing, we found the dose range used led to linear responses when they are measured by IRF-dependent luciferase assay (Fig 4C) and thus chose the dose of 50 μg/mL of 2’3’-cGAMP for the single dose experiments.

### Western blot

Lysis buffer for standard western blot contains 1% NP-40, 50 mM Tris-Cl pH7.4, 150 mM sodium chloride with protease inhibitor (Millipore Sigma, cOmplete ULTRA Tablets EDTA-free Protease inhibitor, Catalog # 6538282001) as described before [26]. For detection of phosphorylated proteins, phosphatase inhibitors are added to the base lysis buffer including 2 mM sodium orthovanadate (Santa Cruz, Catalog # 13721-39-6), 1 mM Phenylmethylsulfonyl fluoride (PMSF) (Millipore-Sigma, Catalog # 52332), and 25 mM β-Glycerophosphate (Sigma-Aldrich, catalog# G9422) as described previously [27]. Antibodies used in this study are β-actin (Sigma-Aldrich, A1978), SAMD9 (Sigma-Aldrich, HPA021319), cGAS (Cell Signaling Technology, 15102S), Phospho-IRF3 (Cell Signaling Technology, 37829S), IRF3 (Cell Signaling Technology, 11904S), FLAG (Sigma-Aldrich, F1804-1MG), RIG-I (Cell Signaling Technology, 3743), MDA5 (Cell Signaling Technology, 5321), V5 (Life Technologies, R960-25). Antibodies for MYXV M040, M038, M062, and M063 are custom made by BIOMATIK Corporation (Delaware, USA).

### DNA pull-down

For dsDNA pull-down experiments, three DNAs are used for the study, VACV70mer [35], HSV60mer [35], and calf thymus dsDNA (Affymetrix, CAS# 9004-34-6). Pre-coupled 5’-biotinylated dsDNA, VACV70mer and HSV60mer, are synthesized by Integrated DNA Technologies (IDT), and DNA pull-down followed the same protocol as described previously [35] with Pierce Strepavidin ultralink resin (Thermo Scientific, Catalog# 53113) and 2 nmol of dsDNA used per experiment. For the dsDNA pull-down experiment using cellulose-conjugated calf thymus dsDNA, we adapted the protocol as described by Kuzuhara et al. [73].

### Flow cytometry and fluorescent microscopy

Differentiated THP1 cells are mock treated, transfected with HT-DNA, infected with wildtype MYXV, or Δ*M062R*. At the 16 hours post-infection or post-treatment, cells were trypsinized for harvesting and fixed in BD Cytofix Fixation Buffer (BD Biosciences, Catalog# 554655) followed by permeabilization with BD Phosflow Perm Buffer III (BD Biosciences, Catalog# 558050). Staining with phosphor-IRF3 is conducted with AF647-phospho-IRF3 antibody (Cell Signaling Technology, 96421S) and samples are analyzed with BD FACS Calibur II. Two independent experiments are performed, and in each experiment triplicate (technical replicate) per sample are analyzed. A successful infection (poxvirus early/late promoter driven GFP or late promoter driven tdTred) and appropriate stages of infection (e.g., late protein synthesis through the detection of tdTred whose expression is driven by poxvirus late promoter) are confirmed through the use of an EVOS FL Auto cell imaging system (Thermo Fisher Scientific).

### Next generation RNA sequencing

THP-1 cells (10^6^ cells per 3.5 cm dish) were differentiated in the presence of phorbol myristic acid (PMA) (Millipore-Sigma) at 50 ng/mL for 48 hours before being mock treated, transfected with ISD [35] at 2 μg per dish using ViaFect (Promega, Madison, WI) at 3 μL per 1 μg of ISD based on manufacturer recommendations, or infected at an moi of 5 for either wildtype (vMyxGFP) or *M062R*-null MYXV [26]. At 8- hour post-transfection with ISD and 8- hour post-viral infection cells were harvested, pelleted by centrifugation and then stored in -80°C until RNA extraction. RNA was extracted using the Quick DNA/RNA Mini-Prep Plus kit (catalog # D7003; Zymo, Irvine, CA, USA) with on-column DNase digestion. Purified RNA was assessed for mass concentration using the Qubit RNA BR Assay kit (catalog # Q10211; Invitrogen, Waltham, MA, USA) and for integrity using the Standard Sensitivity RNA Analysis kit on a Fragment Analyzer capillary electrophoresis system (catalog # DNF-471-0500; Agilent, Santa Clara, CA, USA). A total of 250ng total RNA was used for each sample as input to the TruSeq Stranded Total RNA library prep kit with unique-dual indexing (catalog #s 20020598 & 2002371; Illumina, San Diego, CA, USA). Libraries were assessed for mass using the Qubit 1X dsDNA HS Assay kit (catalog # Q33231; Invitrogen, Waltham, MA, USA), for fragment size using the High Sensitivity NGS Fragment Analysis kit on a Fragment Analyzer capillary electrophoresis system (catalog # DNF-474-0500; Agilent, Santa Clara, CA, USA), and functional validation using the Universal Library Quantification kit (catalog # 07960140001; KAPA, Wilmington, MA, USA).

Validated libraries were adjusted to 3nM before pooling, denaturing, and clustering. Paired-end (2X75) sequencing was performed to an average of 40 million reads per sample on a HiSeq 3000 (Illumina, San Diego, CA, USA).

### Dual RNAseq data processing and Ingenuity Pathway Analysis (IPA)

Raw Illumina binary base call (BCL) files were demultiplexed, adapter trimmed, and transformed to paired-end FASTQ files using bcl2fastq v2.18.0.12 [74]. FastQC v0.11.4 [75] was then used to assess the quality of the FASTQ files. A “hybrid” *H*. *sapiens* (Ensembl GRCh37 build) and Myxoma virus (Lausanne strain, NCBI, NC_001132.2) reference genome was constructed. FASTQ files for each sample were aligned to the hybrid genome using STAR v2.7.6a [76] and run in its two-pass mode. STAR alignment metrics and QualiMap v2.2.1 [77] were used to assess alignment quality.

StringTie v2.1.4 [78] was then used to perform transcriptome reconstruction using each sample’s BAM file. Options were set to only allow the reconstruction and quantification of annotated genes. The gene reference general feature format (GTF/GFF) was produced. Additionally, options were provided to output “Ballgown-ready” files.

For gene-level exploratory data analysis (EDA) and differential expression analysis, StringTie output was imported into DESeQ2 v1.32.0 [79], where p-value and adjusted p-value thresholds were set to 0.05 and 0.1, respectively.

Differential gene expression analysis (e.g., *M062R*-null MYXV infection vs. mock and wildtype MYXV infection vs. mock) was uploaded for IPA to host pathway core analyses, and graphic summary was exported for data presentation.

### Statistical analyses

Graphpad Prism 9.1 was used for statistical analyses. Multiple-group comparison with single variable was performed using One-way ANOVA followed by secondary comparisons (e.g., Tuckey’s multiple comparisons test). Statistical significance was defined as p<0.05.

### Data submission

Sequence data were deposited at the NCBI Gene Expression Omnibus (GEO) (GSE196608) and will be released upon publication of the manuscript.

## Supporting information

S1 Fig Vaccinia virus (VACV) and myxoma virus (MYXV) late proteins are not essential for the inhibition of DNA sensing stimulated IRF-dependent IFN-I induction. **A.** The AraC treatment used in Fig 1A inhibited VACV late gene expression. The wildtype equivalent VACV is engineered to express GFP driven by the poxvirus early/late promoter and tdTomato red driven by the poxvirus late promoter. Before samples described in Fig 1A were harvested for the luciferase assay, they were examined under EVOS microscope using the same filter intensity set up to detect fluorescent expression at a magnification of 10X to confirm the effect of AraC treatment. **B.** The AraC treatment used in Fig 1A significantly inhibited MYXV early/late gene expression. The wildtype MYXV used in Fig 1A and 1B is engineered to express GFP driven by the poxvirus early/late promoter. Before samples described in Fig 1A were harvested for the luciferase assay, they were examined under an EVOS microscope for fluorescent protein expression at a magnification of 10X to confirm the effect of AraC treatment. **C.** Western blot confirms the inhibition of post-replicative gene expression by the same AraC treatment used in Fig 1A. Differentiated THP-1 cells are mock infected or infected with the wildtype MYXV at a moi of 5 for harvesting at the given times. Infected cells were either untreated, treated with AraC at 100 mM, or treated with AraC at 200 mM before, during, and after infection till harvesting. A total protein of 30 μg was loaded per sample lane for the SDS-PAGE separation and western blot. The blot is probed against MYXV intermediate protein M038 (a homolog of VACV I1), late protein SERP1, early/late protein M062, and early/late promoter driven GFP with β-actin as internal loading control.
(TIFF)

S2 Fig DNA pull-down assay shows SAMD9 associated with dsDNA in a cell type independent manner. HeLa cell lysate was incubated with either pre-conjugated streptavidin UltraLink resin with 5’-biotinylated VACV 70mer dsDNA or un-conjugated streptavidin resin alone. After extensive washing, resin-associated content was eluted for western blot analysis by probing for SAMD9. Lane 1: 5’-biotinylated VACV 70mer dsDNA with resin; Lane 2: resin alone.
(TIFF)

S3 Fig The presence of M062 inhibits the association of SAMD9 with dsDNA in a cell type independent manner. With biotinylated VACV70mer dsDNA that were pre-conjugated to streptavidin resin, we infected HeLa cells expressing endogenous SAMD9 with either wildtype MYXV expressing V5 tagged M062 protein or ΔM*062R* MYXV for dsDNA pull-down assay. Proteins associated with DNA were separated on SDS-PAGE for Western Blot probing for SAMD9.
(TIF)

S4 Fig Flow cytometry shows the elevated phosphorylation of IRF3 by ΔM*062R* MYXV infection in differentiated THP1 cells at 16 hours post-infection. Differentiated THP1 cells were mock treated, transfected with HT-DNA, infected with WT or ΔM*062R* MYXV at an moi of 10 for 16 hours before cells were harvested, fixed, permeabilized, and stained with phosphorylated IRF3 antibody that is conjugated with AF647 (Cell signaling, Cat # 96421S). Flow cytometry was analyzed with a BD FACS Calibur, and data analysis was performed using FlowJo. Two independent biological replicates were performed and in each replicate 3 technical replicates were included. Shown is the representative data of one biological replicate. **A.** A representative contour plot with phosphorylated IRF3 (p-IRF3) in y-axis and GFP in x-axis. GFP positive cells from the infection groups were examined for p-IRF3 levels. **B.** A representative histogram of data in “A” is shown. **C.** Comparison of p-IRF3 positive cell percentage among samples shows ΔM*062R* MYXV infected group with significantly elevated p-IRF3 at 16 hours post-infection. The ordinary one-way ANOVA and multiple comparison was performed with statistical significance defined as *p<0.05, **p<0.01, ****p<0.0001.
(TIFF)

S5 Fig Dual RNAseq analyses reveal that infection by ΔM*062R* MYXV results in a unique transcriptomic landscape different from effect by dsDNA alone. **A.** Gene ontology (GO) enrichment analysis (biological process, BP, aspect) was performed using the R library gProfileR (p-value < 0.05). All significantly differentially expressed genes (DEGs) were used as input. Each sample’s value for a given GO:BP was calculated using the mean of the normalized counts for those genes that overlap the given term. Each row’s values were scaled using a z-score method before plotting and ward.D hierarchical clustering was performed using the Euclidean distance measure. Red terms indicate terms enriched among upregulated genes, and green terms represents those that were downregulated. **B.** Top 6 enriched pathways up-regulated by Δ*M062R* MYXV compared to the ISD treatment. The R library ReactomePA was utilized to perform a pathway enrichment analysis. All upregulated DEGs were sent to the library, and the top 6 enriched pathways are displayed (q-value/p.adjust < 0.1). GeneRatio indicates the ratio between the number of overlapping DEGs in the given pathway, and the total number of DEGs. Further, the size of each dot represents the Count, or the total number of overlapping DEGs in a given pathway.
(TIFF)

S6 Fig Overall data distribution from RNAseq. **A.** Venn diagram of host genes identified as being differentially regulated in ISD, ΔM*062R*, and wildtype MYXV groups compared to mock treated cells. Gene lists were generated by performing differential gene expression analysis using the R library DESeQ2, and only those genes whose adjusted-p-value was less than 0.1 were included in the analysis. **B.** Venn diagram of MYXV specific genes detected in dual RNAseq analyses. Almost all viral genes expressed by wildtype MYXV at 8 h were detected in ΔM*062R* infection.
(TIFF)

S7 Fig Raw data alignment of MYXV specific reads from Δ*M062R* and wildtype MYXV infection showed that only the *M062R* gene was absent in samples infected with the Δ*M062R* MYXV. Alignment files (i.e., BAM) from the first replicate from each of the Δ*M062R* and wildtype MYXV infection, were uploaded to igv.org/app/ for visualization. The dual genome reference was also uploaded and only the base pairs corresponding to the *M062R* gene were displayed.
(TIFF)
