# Supplementary figures and images for: Myxoma virus lacking the host range determinant M062 stimulates cGAS-dependent type 1 interferon response and unique transcriptomic changes in human monocytes/macrophages
Source: PLoS Pathog. 2022 Sep 14;18(9):e1010316. doi: 10.1371/journal.ppat.1010316 (PMC9473615; doi:10.1371/journal.ppat.1010316)

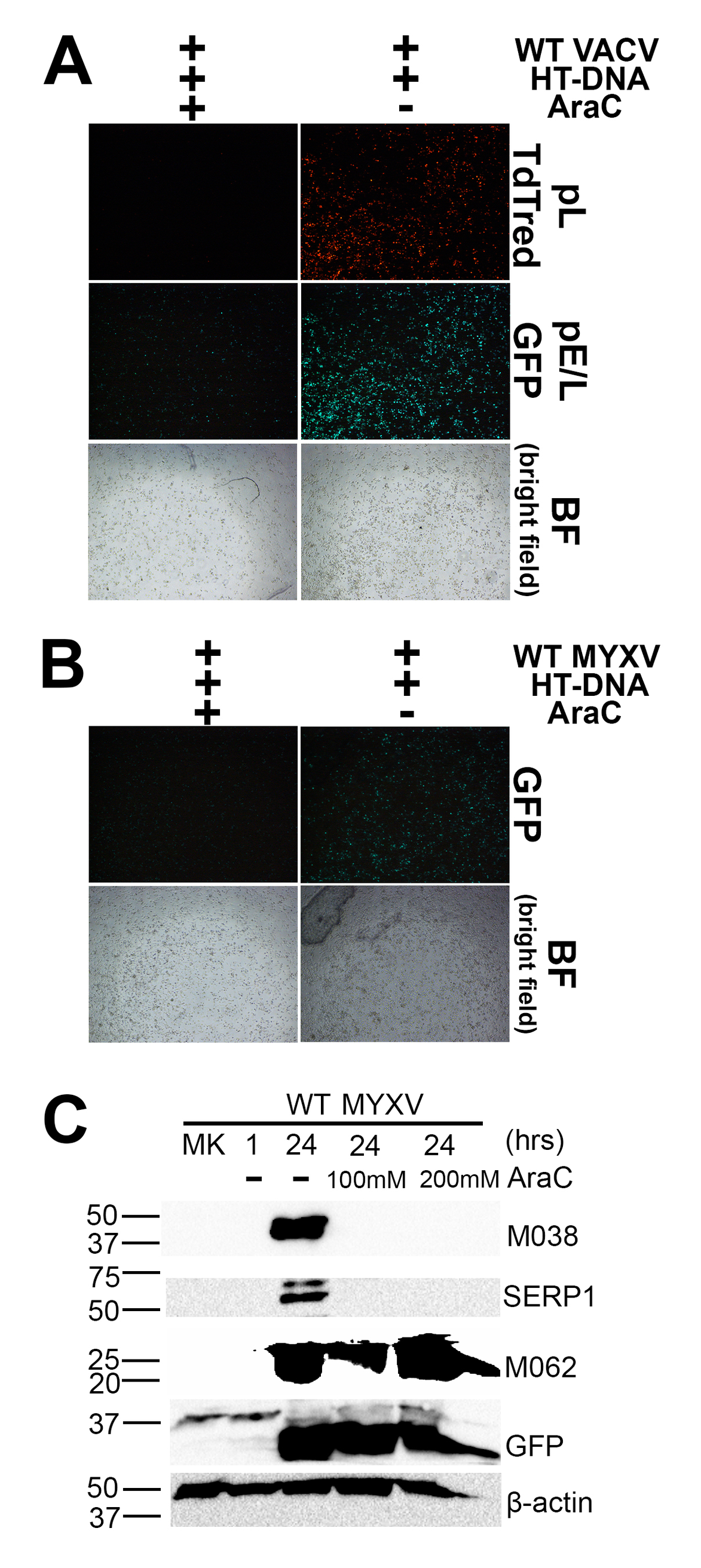

Supplement: S1 Fig — A. The AraC treatment used in Fig 1A inhibited VACV late gene expression. The wildtype equivalent VACV is engineered to express GFP driven by the poxvirus early/late promoter and tdTomato red driven by the poxvirus late promoter. Before samples described in Fig 1A were harvested for the luciferase assay, they were examined under EVOS microscope using the same filter intensity set up to detect fluorescent expression at a magnification of 10X to confirm the effect of AraC treatment. B. The AraC treatment used in Fig 1A significantly inhibited MYXV early/late gene expression. The wildtype MYXV used in Fig 1A and 1B is engineered to express GFP driven by the poxvirus early/late promoter. Before samples described in Fig 1A were harvested for the luciferase assay, they were examined under an EVOS microscope for fluorescent protein expression at a magnification of 10X to confirm the effect of AraC treatment. C. Western blot confirms the inhibition of post-replicative gene expression by the same AraC treatment used in Fig 1A. Differentiated THP-1 cells are mock infected or infected with the wildtype MYXV at a moi of 5 for harvesting at the given times. Infected cells were either untreated, treated with AraC at 100 mM, or treated with AraC at 200 mM before, during, and after infection till harvesting. A total protein of 30 μg was loaded per sample lane for the SDS-PAGE separation and western blot. The blot is probed against MYXV intermediate protein M038 (a homolog of VACV I1), late protein SERP1, early/late protein M062, and early/late promoter driven GFP with β-actin as internal loading control. (TIFF) [file ppat.1010316.s001.tiff]

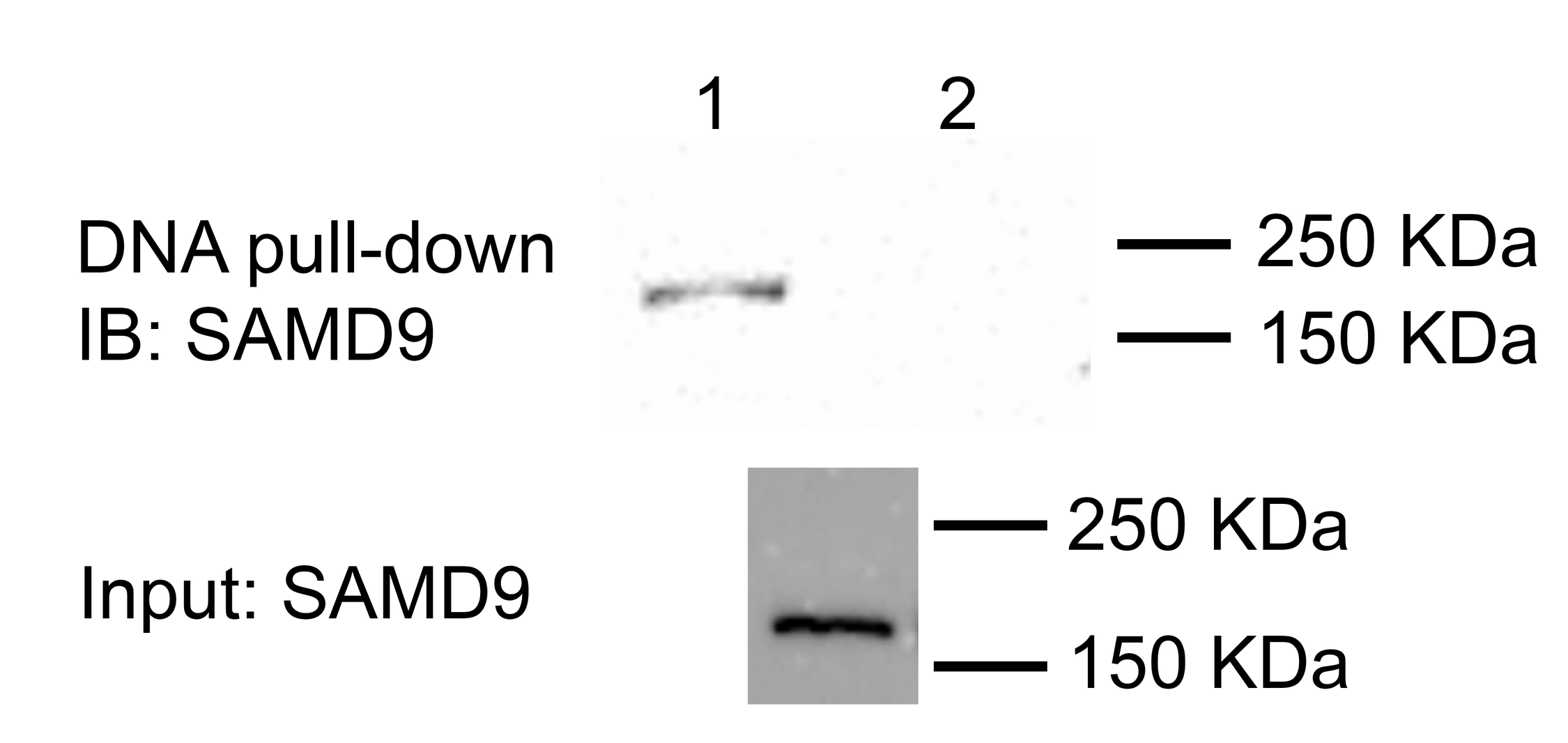

Supplement: S2 Fig — HeLa cell lysate was incubated with either pre-conjugated streptavidin UltraLink resin with 5’-biotinylated VACV 70mer dsDNA or un-conjugated streptavidin resin alone. After extensive washing, resin-associated content was eluted for western blot analysis by probing for SAMD9. Lane 1: 5’-biotinylated VACV 70mer dsDNA with resin; Lane 2: resin alone. (TIFF) [file ppat.1010316.s002.tiff]

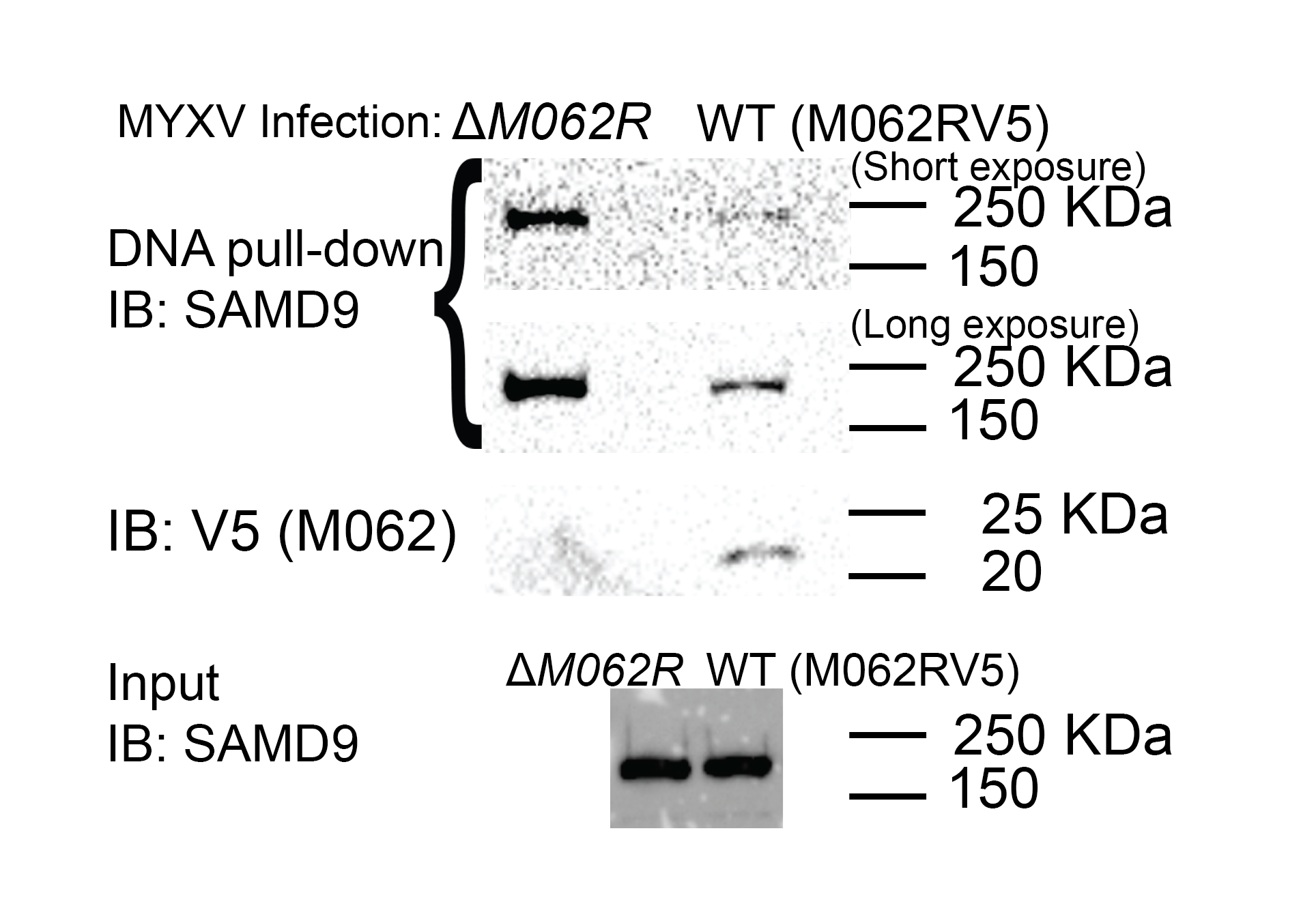

Supplement: S3 Fig — With biotinylated VACV70mer dsDNA that were pre-conjugated to streptavidin resin, we infected HeLa cells expressing endogenous SAMD9 with either wildtype MYXV expressing V5 tagged M062 protein or ΔM062R MYXV for dsDNA pull-down assay. Proteins associated with DNA were separated on SDS-PAGE for Western Blot probing for SAMD9. (TIF) [file ppat.1010316.s003.tif]

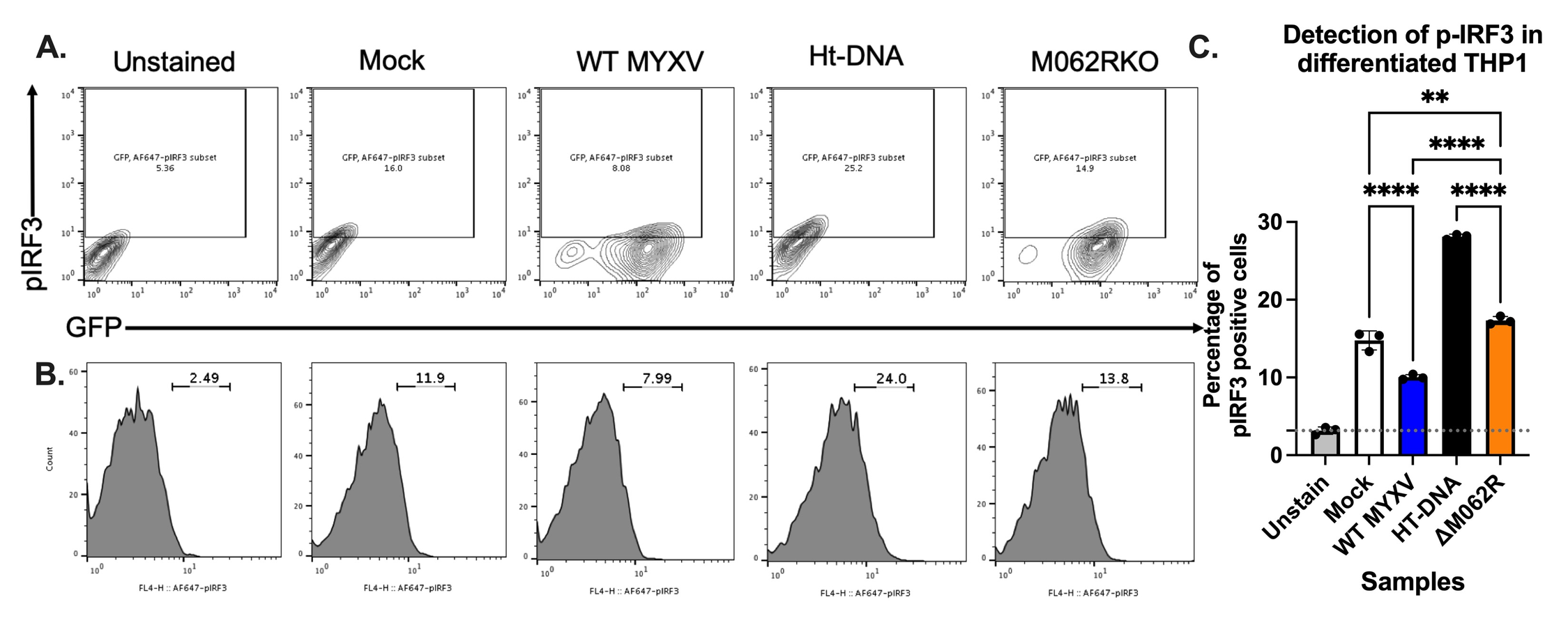

Supplement: S4 Fig — Differentiated THP1 cells were mock treated, transfected with HT-DNA, infected with WT or ΔM062R MYXV at an moi of 10 for 16 hours before cells were harvested, fixed, permeabilized, and stained with phosphorylated IRF3 antibody that is conjugated with AF647 (Cell signaling, Cat # 96421S). Flow cytometry was analyzed with a BD FACS Calibur, and data analysis was performed using FlowJo. Two independent biological replicates were performed and in each replicate 3 technical replicates were included. Shown is the representative data of one biological replicate. A. A representative contour plot with phosphorylated IRF3 (p-IRF3) in y-axis and GFP in x-axis. GFP positive cells from the infection groups were examined for p-IRF3 levels. B. A representative histogram of data in “A” is shown. C. Comparison of p-IRF3 positive cell percentage among samples shows ΔM062R MYXV infected group with significantly elevated p-IRF3 at 16 hours post-infection. The ordinary one-way ANOVA and multiple comparison was performed with statistical significance defined as *p<0.05, **p<0.01, ****p<0.0001. (TIFF) [file ppat.1010316.s004.tiff]

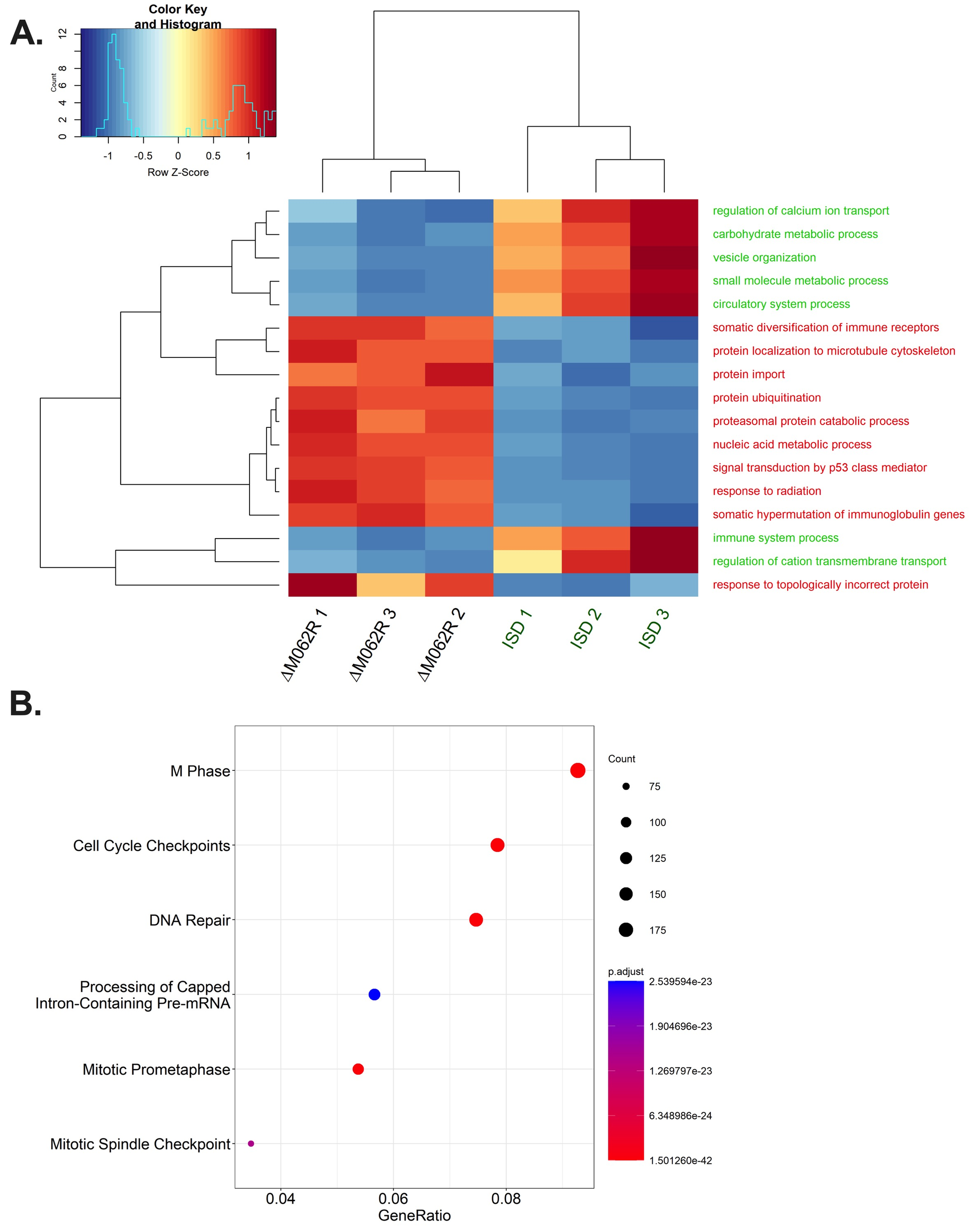

Supplement: S5 Fig — A. Gene ontology (GO) enrichment analysis (biological process, BP, aspect) was performed using the R library gProfileR (p-value < 0.05). All significantly differentially expressed genes (DEGs) were used as input. Each sample’s value for a given GO:BP was calculated using the mean of the normalized counts for those genes that overlap the given term. Each row’s values were scaled using a z-score method before plotting and ward.D hierarchical clustering was performed using the Euclidean distance measure. Red terms indicate terms enriched among upregulated genes, and green terms represents those that were downregulated. B. Top 6 enriched pathways up-regulated by ΔM062R MYXV compared to the ISD treatment. The R library ReactomePA was utilized to perform a pathway enrichment analysis. All upregulated DEGs were sent to the library, and the top 6 enriched pathways are displayed (q-value/p.adjust < 0.1). GeneRatio indicates the ratio between the number of overlapping DEGs in the given pathway, and the total number of DEGs. Further, the size of each dot represents the Count, or the total number of overlapping DEGs in a given pathway. (TIFF) [file ppat.1010316.s005.tiff]

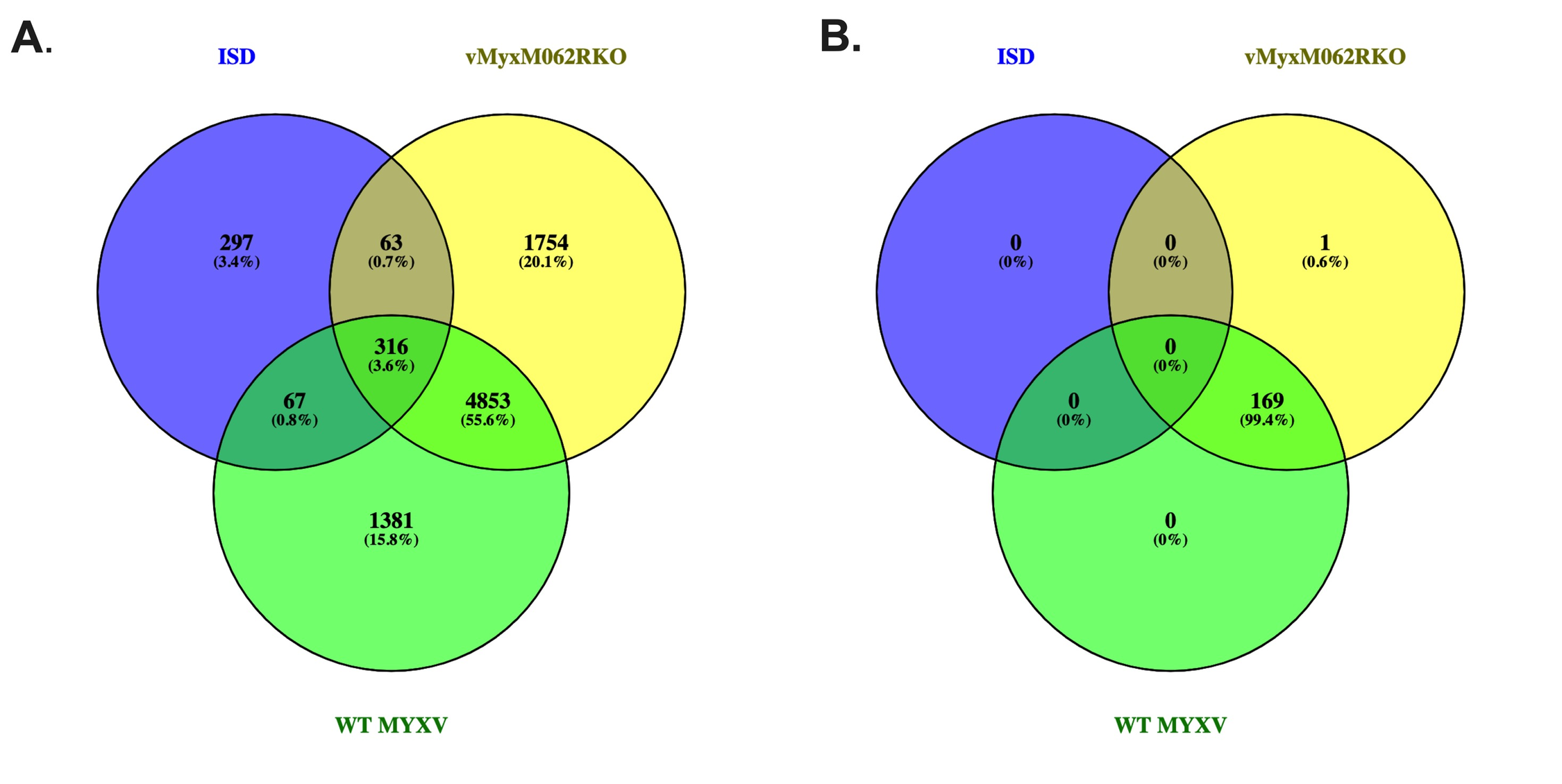

Supplement: S6 Fig — A. Venn diagram of host genes identified as being differentially regulated in ISD, ΔM062R, and wildtype MYXV groups compared to mock treated cells. Gene lists were generated by performing differential gene expression analysis using the R library DESeQ2, and only those genes whose adjusted-p-value was less than 0.1 were included in the analysis. B. Venn diagram of MYXV specific genes detected in dual RNAseq analyses. Almost all viral genes expressed by wildtype MYXV at 8 h were detected in ΔM062R infection. (TIFF) [file ppat.1010316.s006.tiff]

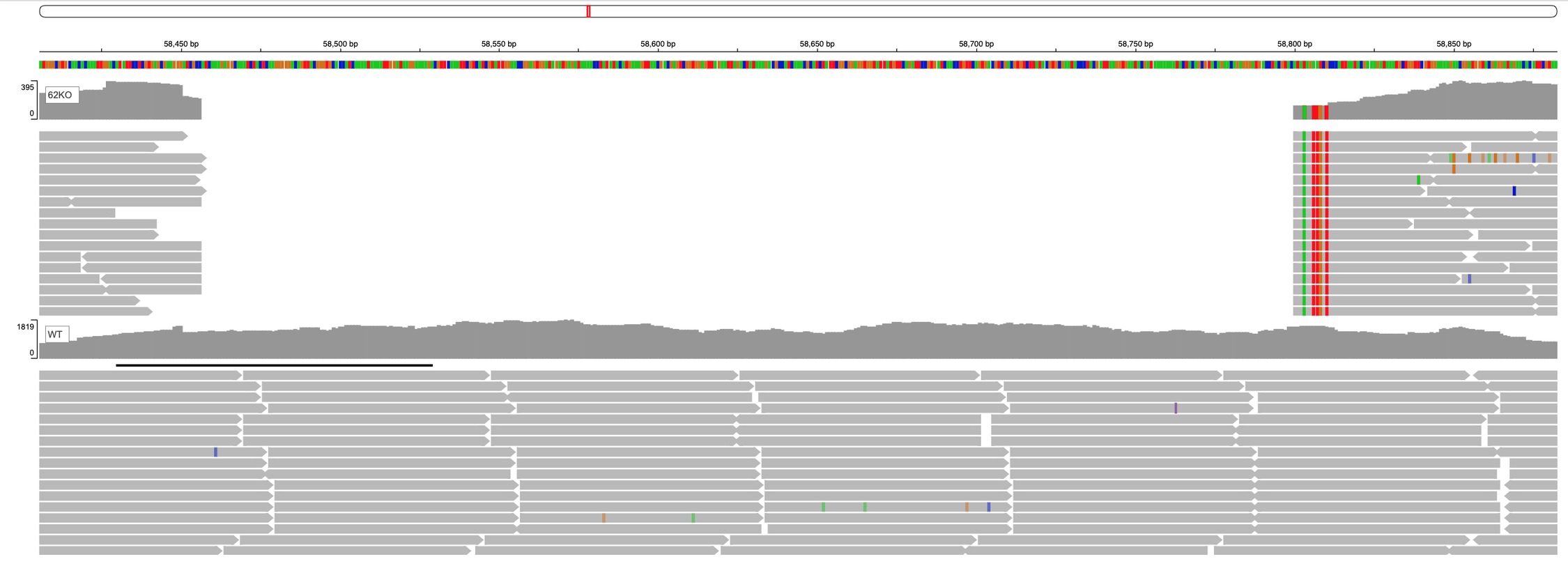

Supplement: S7 Fig — Alignment files (i.e., BAM) from the first replicate from each of the ΔM062R and wildtype MYXV infection, were uploaded to igv.org/app/ for visualization. The dual genome reference was also uploaded and only the base pairs corresponding to the M062R gene were displayed. (TIFF) [file ppat.1010316.s007.tiff]
